# Modeling the past, present, and future distributions of endangered white abalone (*Haliotis sorenseni*) to inform recovery efforts in California

**DOI:** 10.1371/journal.pone.0259716

**Published:** 2021-11-17

**Authors:** Jordan DiNardo, Kevin L. Stierhoff, Brice X. Semmens

**Affiliations:** 1 University of California San Diego: Scripps Institution of Oceanography, La Jolla, California, United States of America; 2 Southwest Fisheries Science Center, National Marine Fisheries Service, La Jolla, California, United States of America; University of Nevada, Reno, UNITED STATES

## Abstract

White abalone (*Haliotis sorenseni*) was once commonly found in coastal waters of the Southern California Bight (SCB) and south to Punta Abreojos, Baja California, Mexico. During the 1970s, white abalone supported a commercial fishery, which reduced the population and resulted in the closure of the fishery in 1996. When population levels continued to decline, National Marine Fisheries Service (NMFS) listed the species as endangered under the Endangered Species Act. The California Department of Fish and Wildlife and NMFS began surveying the wild populations, propagating specimens in captivity, and protecting its seabed habitat. We modeled coarse-scale (17 x 17 km) historical (using fishery-dependent data [1955–1996]) and contemporary (using fishery-independent data [1996–2017]) distributions of white abalone throughout its historical domain using random forests and maximum entropy (MaxEnt), respectively, and its fine-scale (10 x 10 m) contemporary distribution (fishery-independent data) using MaxEnt. We also investigated potential outplanting habitat farther north under two scenarios of future climate conditions. The coarse-scale models identified potential regions to focus outplanting efforts within SCB while fine-scale models can inform population monitoring and outplanting activities in these particular areas. These models predict that areas north of Point Conception may become candidate outplant sites as seawater temperatures continue to rise in the future due to climate change. Collectively, these results provide guidance on the design and potential locations for experimental outplanting at such locations to ultimately improve methods and success of recovery efforts.

## Introduction

White abalone (*Haliotis sorenseni*) is a marine gastropod once commonly found along the west coast of North America, between Point Conception, California, and Punta Abreojos, Baja California, Mexico, including the California Channel Islands. Thought to be the deepest-living of the six west coast abalone species, white abalone occurred in high numbers between 30–70 m of water [1, 2], where brown algae species, such as *Laminaria farlowii* and *Agarum fimbriatum*, and a variety of red algae species are common and provide food for white abalone [2, 3].

Abalone have been harvested in California for 7,500 years [4]. In 1955, the California Department of Fish and Wildlife (CDFW; formerly California Department of Fish and Game,CDFG), officially named white abalone a “species to be harvested” (commercially). In the late 1960s, reported white abalone landings increased as shallower abalone species decreased [3]. Landings of white abalone peaked in 1972 (65 metric tons), predominantly taken from San Clemente Island, and then declined. Over 95% of the reported commercial white abalone catch occurred in nine years (1969–1977). By 1978, catches of white abalone became so rare that CDFW eliminated mandatory reporting on fishing receipts [3].

Due to overfishing, CDFW closed the commercial and recreational fisheries for white abalone in 1996, along with those for green abalone (*H*. *fulgens*) and pink abalone (*H*. *corrugata*) [3, 5]. Despite these closures, white abalone populations continued to decline and in 2001 the species became the first marine invertebrate to be listed as endangered under the Endangered Species Act (ESA). In 2008, the National Marine Fisheries Service (NMFS) published a White Abalone Recovery Plan that identified goals and strategies to recover the species and delist it from the ESA, including field monitoring and captive propagation for enhancement of wild populations [6]. NMFS conducts surveys using a remotely operated vehicle (ROV), and more recently SCUBA, to monitor white abalone populations, characterize their seabed and oceanographic habitats, and collect broodstock for a captive-breeding program. Due to its lack of recovery and imminent extinction risk [7], NMFS designated white abalone as a “Species in the Spotlight” in 2016, compelling efforts to identify habitats that can support the survival and growth of outplanted white abalone.

Since environmental conditions largely determine the spatial and temporal distributions of biota, species distribution models (SDMs) are used to define suites of environmental characteristics that are suitable for a given species. In the field of conservation biology, SDMs are often used to inform outplanting efforts [8–10]. This study developed SDMs to support outplanting strategies of white abalone in the Southern California Bight (SCB) using commercial landings data and observations from fishery-independent surveys during two different periods. When applicable, we modeled habitat suitability at two spatial scales: coarse-scale (17 x 17 km) and fine-scale (10 x 10 m). Coarse-scale models resolve the CDFW fishing blocks (10 X 10 arcminutes), spanning the area of the species’ distributional range. Fine-scale models resolve areas pertinent to abalone presence and movement of individuals. Finally, we investigated potential habitat north of the species’ historical range under environmental scenarios expected to result from climate change.

## Materials and methods

### Study area

The study area for the coarse-scale models was the SCB, from Point Conception, California, to the California-Mexico border, including the eight Channel Islands [11]. For fine-scale models, this area was divided into five areas including: San Clemente Island, Tanner and Cortes Banks, Santa Catalina and Santa Barbara Islands, San Diego, and Palos Verdes. These study areas reflect patterns in white abalone landings from CDFW commercial catch data [12], observations from fishery-independent surveys [1, 2, 7, 13, 14, M. Neuman, unpublished data], and the spatial coverage of the available fine-scale environment data (see below).

### Species distribution data

#### Coarse-scale models

The fishery-dependent data were white abalone commercial landings (pounds per fishing block and year) from CDFW fishing receipts from 1955 to 1996. To facilitate modeling, we converted white abalone landings to numbers of individuals using the average adult weight (1.7 pounds; I. Taniguchi, personal communication), which resulted in a total catch of 917,728 individuals with over 80% from the San Clemente Island area [3]). Assuming fishermen conducted an exhaustive search for white abalone within its historical range, we assumed areas with no catch to be void or to have negligible numbers of white abalone. Because these data span the duration of the fishery, it is also assumed that the catches approximate the relative abundance and distribution of the population, which are suitable for modeling with the regressional ecological modeling methods used here.

Fishery-independent data included georeferenced observations of white abalone from surveys conducted in the SCB between 1976 and 2017 using SCUBA (1976, 1996, 2010–2017) [1, 13, 15, M. Neuman, personal communication], manned submersibles (1998–1999) [14], and remotely operated vehicles (ROVs) (2002–2017) [2, 7]. A combined 606 white abalone observations motivated the use of presence-background modeling methods (described in *Species Distribution Modeling*). However, to compare and evaluate relative habitat suitability at a coarse-scale across the historical and contemporary periods, we also analyzed these data on the scale of the fishery-dependent observations. Since presence-background models are sensitive to redundant observations (more than one individual located within the same 10 x 10 m block), we constrained these data to one observation per block, which resulted in 21 blocks with fishery-independent observations.

#### Fine-scale models

We used fishery-independent observations from 1993–2017, when ± 1–6 m spatial resolution was most consistent. To avoid model mis-specification, we removed redundant white abalone observations within 5m radius, resulting in 26 observations at San Clemente Island, 359 at Tanner and Cortes Banks; 16 at Santa Barbara Island and Santa Catalina Island; 19 at San Diego; and 14 at Palos Verdes.

### Environmental data for spatially and temporally varying scenarios

#### Coarse-scale models

For coarse-scale models and projections in time and space, we derived seabed depth (m), seabed slope (°), and length of coastline (km) from the CDFW Marine Region Geographic Information System (GIS) Lab [16]. We derived mean sea surface temperature (SST; °C) from California Cooperative Oceanic Fisheries Investigation (CalCOFI) survey data [17]. We derived mean SST data forecasts for 2050 and 2100 from Bio-ORACLE [18, 19], which are from four representative concentration pathway (RCP) scenarios of varying CO_2_ concentration levels: RCP2.6, a peak-and-decline scenario resulting in low concentration levels of CO_2_ by 2100; RCP4.5 and RCP6.0, where CO_2_ levels stabilize through time; and RCP8.5 where CO_2_ concentration increases continuously through time, resulting in high concentrations of greenhouse gases. These variables are known to influence the distribution and survival of white abalone [2, 3, 15, 20, 21]. Pearson correlation coefficients for each model run indicated whether these coarse-scale environmental variables co-vary on either spatial scale. In such cases, we omitted the less informative variable (see S1–S3 Files for details).

#### Fine-scale models

For fine-scale models, we obtained seabed depth (m), seabed slope (°), and vector ruggedness measure (VRM) from the Seafloor Mapping Lab at California State University Monterey Bay [22]. We derived kelp persistence indices (a measure of the binary maximum likelihood of *Macrocystis pyrifera* presence through time) from data obtained from the Santa Barbara Coastal Long-Term Ecological Research Project [23]. We derived indices of predator diversity and of abundance indices of predators (i.e., California two-spot octopus, *Octopus bimaculoides*), and competitors (i.e., red urchin, *Strongylocentrotus franciscanus*, and purple urchin, *Strongylocentrotus purpuratus*) from data obtained from Reef Environmental Education Foundation [24]. Not all of these data were available for each fine-scale area. For example, models for San Clemente Island only included seabed depth, seabed slope, and VRM because the other variables did not overlap with white abalone observations at that location. See the S1–S3 Files for more detailed definitions and methods used to derive these variables and for diagnostic tests for each model scale.

### Species distribution modeling

We modeled the historical and contemporary distributions of white abalone within the SCB at the coarse-scale using random forests and MaxEnt, respectively. Despite the challenges of comparing results from differing model techniques, these two modeling techniques consider the respective strengths and limitations of each dataset most effectively and relative comparisons of results offer valuable insight of suitable habitat during their respective periods. We used these models to project habitat suitability under differing RCP scenarios throughout California, resulting in 16 future scenario combinations (2 models x 2 future periods x 4 scenarios). These projections helped to assess the stability of suitable habitat for white abalone and identify potential habitat outside of the species’ known geographic range under various future climate scenarios. Using MaxEnt, we also modeled the contemporary distribution of white abalone within five study areas in the SCB. For each respective model scenario, we computed the mean and 95% confidence intervals (CI) of habitat suitability.

We used random forests, a non-parametric machine learning modeling technique, to predict fishery-dependent catch landings. In brief, random forests are an ensemble classification and regression tree analysis that provide a mean prediction from an ensemble of individual trees; see Breiman [25] for a detailed overview. To stabilize the error of the model, we grew 10,000 trees using the ‘randomForest’ package [26] in R [27]. To alleviate the zero-inflation and skewness of the fishery-dependent data, we log-transformed catch data. The outputs from the random forest analyses represent the expected catch, which we converted to relative catch. We split fishery-dependent data into 70% training and 30% test data to calculate the percent variability explained and root mean square error (RMSE). Since the fishery effectively fished the population near extinction, we assumed relative catch served as a proxy for relative abundance, and ultimately a metric of relative habitat suitability ranging from zero (unstable) to 1.0 (optimal).

We also used MaxEnt, another machine learning method and one of the few methods capable of using presence-only data to model a species’ distribution; see Elith et al. [28] for a detailed explanation of MaxEnt. Due to low numbers of fishery-independent observations, we used all records as training data. Using the ‘dismo’ [29] package in R [27], ‘maxent’ generated the mean relative probability of presence (averaged over 100 model runs), ranging from zero to 0.75 (maximum output of relative habitat suitability from the model), which was a metric of relative habitat suitability to compare to results from the regressional random forest analysis. Since model performance is at least partially reliant on model tuning parameters (regularization multiplier and feature classes) [30, 31], we compared a range of regularization levels (1–10) and feature classes (linear and quadratic) using small sample size corrected Akaike information criterion (AICc) from the ENMeval package in R [32]. In each case, we present model results based on a the top performing models based on AICc. In efforts to avoid overfitting, we limited modeling to linear or quadratic feature types (excluding product, threshold, hinge feature types, which are known to allow for more complex relationships between environmental variables and species observations).

Area under the curve (AUC) of the receiver-operating characteristic is typically used to validate presence-absence and presence-background models. Since AUC is especially sensitive to low species prevalence [33], which was the case here, we used a null model approach for significance testing of Maxent models (coarse-scale and fine-scale). This approach tests the AUC values of the MaxEnt models against a null distribution of expected AUC values based on random sampling of data [34].

To assess the relative importance of each environmental variable in explaining white abalone distribution, we compared corresponding variables’ percent increase in mean square error (MSE) for fishery-dependent models using random forests and average percent contribution (APC) for fishery-independent models using MaxEnt. We also used APC to assess relative importance of environmental variables for fine-scale models and calculated the overall mean percent contribution of each variable’s APC across all study areas. Lastly, we developed response curves for each environmental variable to examine their marginal effects on the prediction of relative habitat suitability.

## Results

### Coarse-scale models

**Fig 1:**
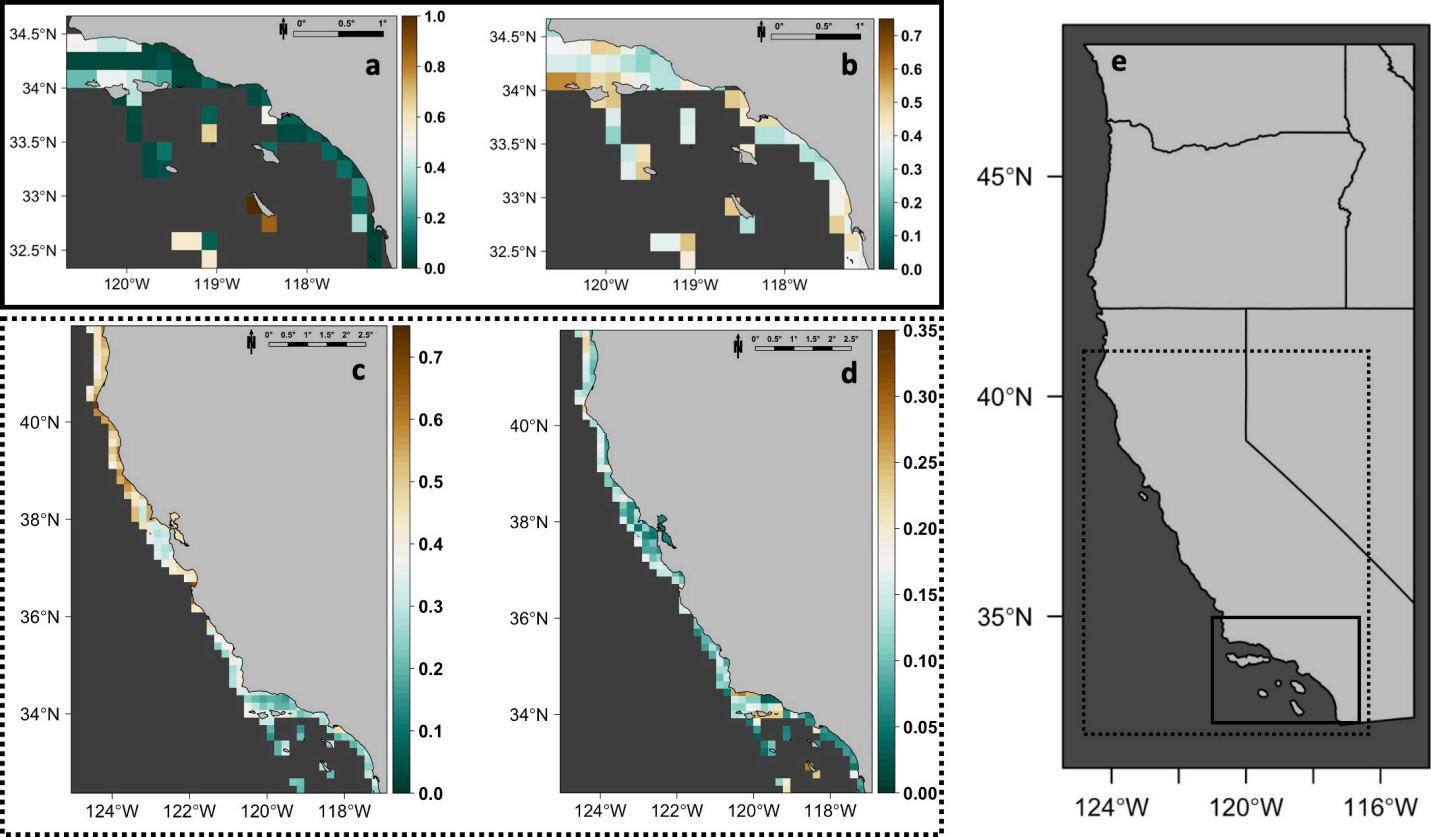
Predicted and projected white abalone relative mean habitat suitability based on historical and contemporary distributions. Upper panels represent suitability in the Southern California Bight (outlined in the solid bounding box of panel f) based on fishery-dependent (a) and fishery-independent (b) models during their respective time periods. Lower panels represent future predictions of relative mean habitat suitability throughout California waters (outline in the dotted bounding box of panel f) in 2050 under the RCP 2.6 scenario, based on the fishery-dependent (c) and fishery-independent models (d). Suitability interpreted from log-transformed relative mean abundance using random forest for the fishery-dependent model and relative probability of presence using MaxEnt for the fishery-independent model where unsuitable = zero and most suitable = 1 (or for fishery-independent model most suitable = 0.75). Relative mean habitat suitability is visualized for fishing blocks ≤ 500 m in depth. Note differences in suitability scales across predicted and projected panels.

**Fig 2:**
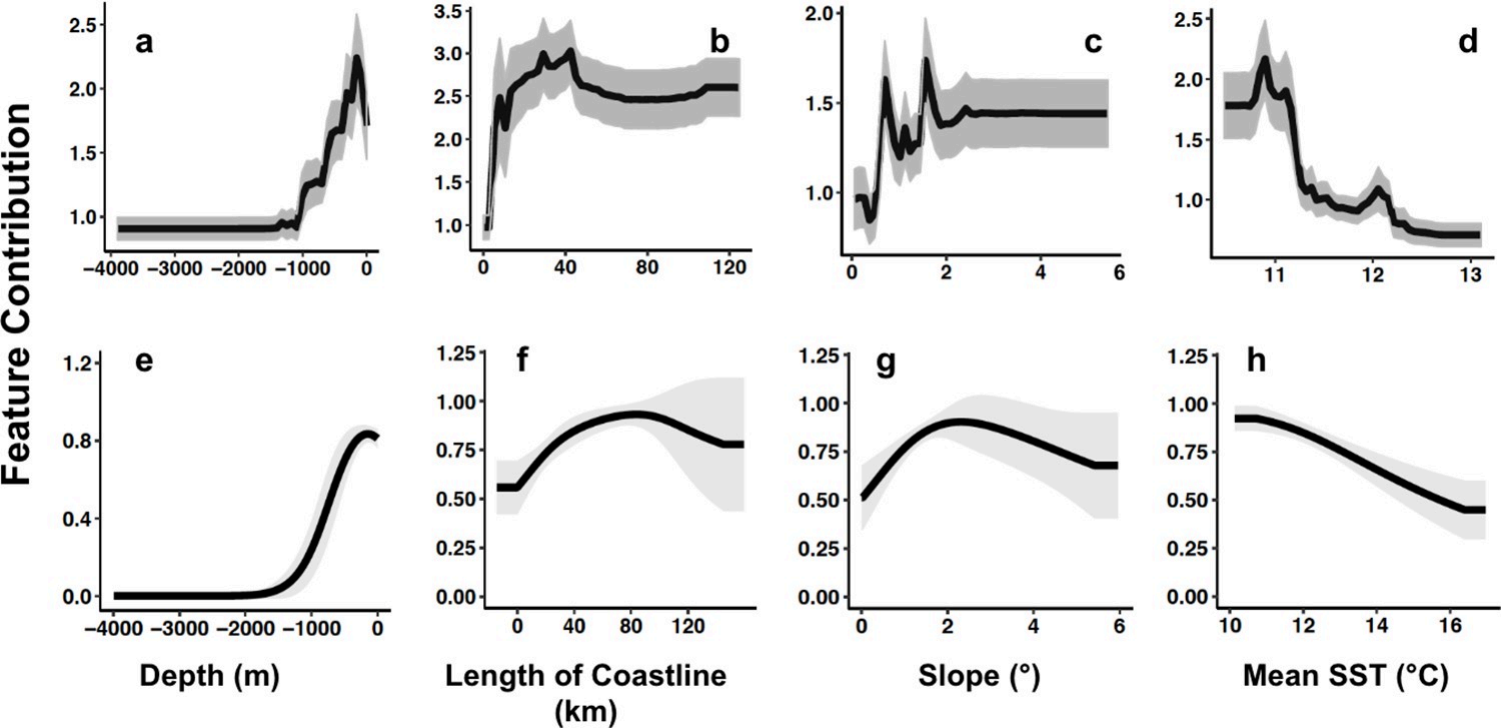
Response curves for broad-scale environmental variables. Response curves for the fishery-dependent model (a-d) are developed from random forest and response curves for the fishery-independent models (e-h) are developed from MaxEnt. The black line represents the mean response of 100 model runs and the grey shaded region represents one SD of the mean.

The coarse-scale fishery-dependent random forest model identified a limited amount of relative suitable habitat (relative suitability ≥ 0.5) for white abalone (Fig 1A). In general, San Clemente Island exhibited the highest relative habitat suitability within the SCB (habitat suitability > 0.8). Tanner Bank and Cortes Bank were less suitable (habitat suitability ranged from 0.5–0.6), followed by the northern Channel Islands (Santa Rosa, San Miguel, Santa Cruz, and Anacapa) and areas along the mainland coast of southern California (habitat suitability ranged from 0.3–0.5) (Fig 1A). The fishery-dependent models identified a majority of the southern California coast as unsuitable, except for Palos Verdes and the northern region between Point Conception and Santa Barbara, California. The coarse-scale fishery-dependent model explained 22.82% of variability in the data and obtained a RMSE of 2.751. Seabed depth exhibited the highest mean percent increase in MSE (71.22%, interquartile range (IQR) = 36.58%), followed by length of coastline (70.03%, IQR = 42.65%), mean SST (63.65, IQR = 43.36%), and seabed slope (41.09, IQR = 34.35%). The marginal effects of each variable indicated that relative habitat suitability was highest in areas of shallow seabed depths (0–500 m), intermediate lengths of coastline (10–50 km), low seabed slope (1–2°), and cooler mean SST (10.5–11°C) (Fig 2A–2D). The fishery-independent MaxEnt model identified more areas with suitable habitat (Fig 1B). The northern Channel Islands, specifically San Miguel, exhibited the highest relative habitat suitability (habitat suitability >0.6). Habitat suitability was moderately high at San Nicolas Island, Santa Catalina Island, northern San Clemente Island, and at Tanner and Cortes Banks (habitat suitability ranged from 0.3–0.5). Similar to the fishery-dependent model, the fishery-independent model identified a majority of the Southern California coast as unsuitable, except near San Diego, California, Palos Verdes, and parts of the northern region between Gaviota and Isla Vista, California (habitat suitability ranged from 0.3–0.5). The fishery-independent model had a training AUC value of 0.880. When compared to the null model (upper bound of 95% CI of AUC value = 0.875), the fishery-independent model was significantly better in predicting suitable habitat than that predicted by chance (*p* < 0.001). Seabed depth had the highest APC (60.40%, IQR = 14.82%), followed by length of coastline (25.50%, IQR = 62.04%), seabed slope (7.40%, IQR = 22.87%), and mean SST (6.70%, IQR = 19.07%). The marginal effects of each environmental variable denoted similar influences on relative habitat suitability with the coarse-scale fishery-dependent model, although definitions of suitable habitat were less specific than that for the fishery-independent model (Fig 2E–2H).

Within the SCB, habitat suitability projections for both the coarse-scale fishery-dependent and fishery-independent models decreased in suitability from that exhibited during their respective periods (Fig 1C and 1D; and S1–S3 Files). While relative habitat suitability decreased into the future under worsening RCP scenarios, a majority of the areas that were identified as having the highest relative habitat suitability in the contemporary models also occupied the highest relative suitable habitat in the future, especially for fishery-dependent model projections. Projections of the coarse-scale fishery-independent model identified areas north of Point Conception, California, (specifically around Cape Mendocino, Point Arena, Point Reyes, and Monterey Bay) to have the highest relative habitat suitability in the future.

### Fine-scale models

**Fig 3:**
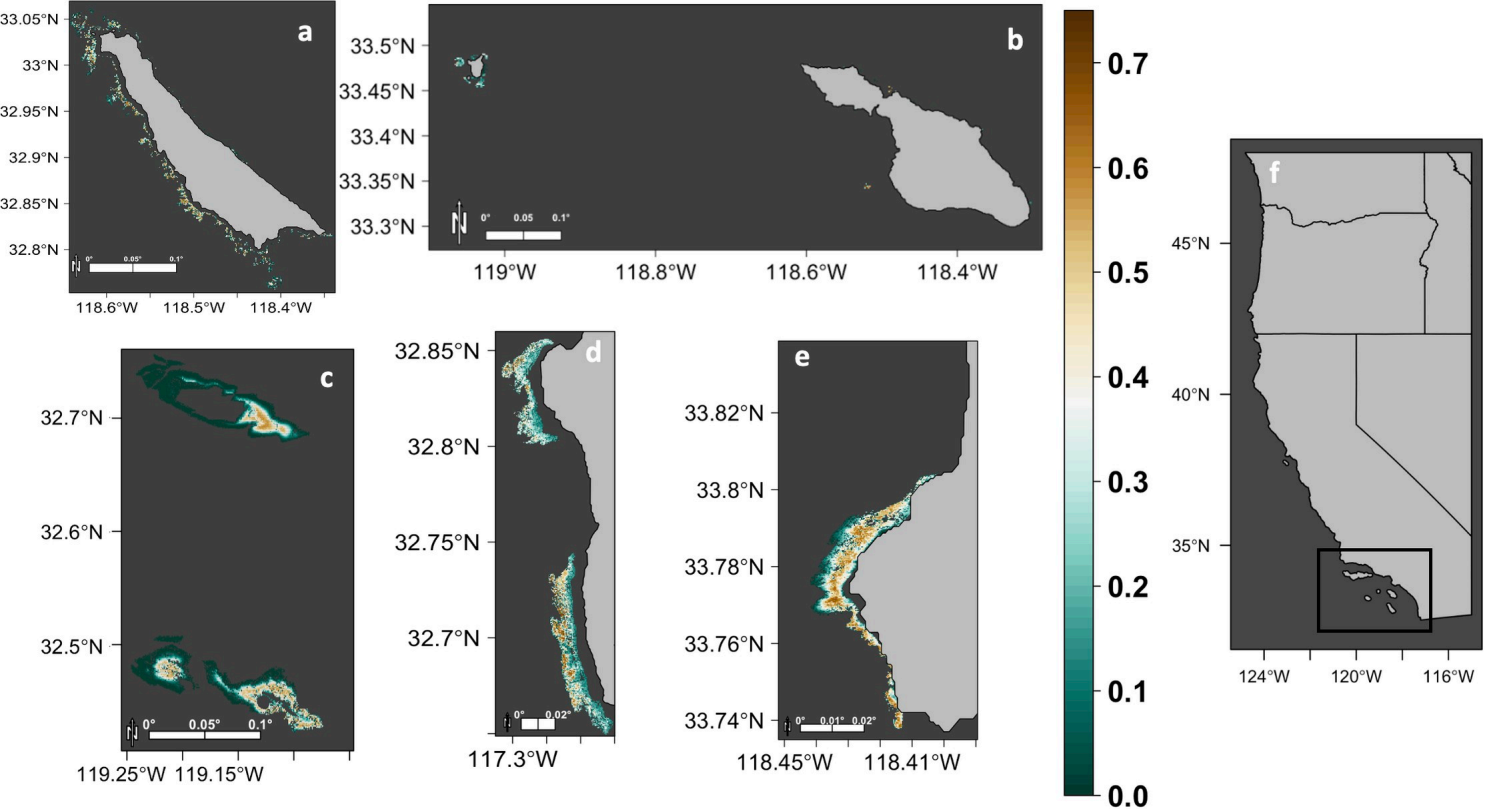
Predicted relative mean probability of white abalone presence (relative mean habitat suitability). Predicted relative mean probability of white abalone presence in five areas located within the Southern California Bight (f), including San Clemente Island (a), Santa Catalina and Santa Barbara Islands (b), Tanner and Cortes Banks (c), San Diego (d), and Palos Verdes (e). Each panel represents the mean probability of abalone presence averaged over 100 model runs, ranging from unsuitable (0) to most suitable (0.7).

Based on fine-scale, fishery-independent models, the most suitable habitat occurred in areas with hard substrate between 30 and 70 m depth at offshore sites (San Clemente Island, Santa Catalina Island, Santa Barbara Island, Tanner Bank, and Cortes Bank; Fig 3A–3C); and between the depths of 10 and 30 m along the mainland coast in southern California (Fig 3D and 3E). The model for each study area had an average AUC value >0.7 (San Clemente Island: 0.776, Tanner and Cortes Banks: 0.805, Santa Catalina and Santa Barbara Islands: 0.937, San Diego 0.770, and Palos Verdes: 0.904). When compared to their respective null models (95% CI upper bounds of AUC were 0.770, 0.803, 0.934, 0.764, 0.898 for San Clemente Island, Tanner and Cortes Banks, Santa Catalina and Santa Barbara Islands, San Diego, and Palos Verdes, respectively), all models were significantly better at predicting relative habitat suitability than that predicted by chance (*p* values<0.001).

Seabed depth had the highest overall mean percent contribution (57.82%, IQR = 73.67%), followed by kelp persistence index (36.29%, IQR = 35.05%), California two-spot octopus abundance index (22.14%, IQR = 0), seabed slope (10.64%, IQR = 26.33%), predator diversity index (10.35%, IQR = 0), VRM (9.38%, IQR = 15.57%) and urchin abundance index (5.67%, IQR = 0). Response curves of these variables (S1–S3 Files) indicated that habitat suitability was highest at depths between 30 and 70 m for offshore sites and 10–30 m for areas along the southern California coast. Relative habitat suitability decreased as VRM, seabed slope, predator diversity index, and urchin abundance indices increased. Relative habitat suitability was generally high across the range of two-spot octopus abundance indices. Kelp persistence exhibited conflicting results between the two study areas where it was included; habitat suitability increased with the kelp persistence index at San Diego, but decreased with increasing kelp persistence at Palos Verdes (S1–S3 Files).

## Discussion

### Model outputs and evaluation

#### Coarse-scale models

Coarse-scale fishery-dependent and fishery-independent models reflected differences in historical and contemporary white abalone distributions. Although the rankings of importance and marginal effects of each environmental variable did not differ considerably between coarse-scale models, predicted relative habitat suitability within the Southern California Bight (SCB) did. An underlying factor driving this difference in the model outcomes is the differing modeling periods and corresponding species data properties. Inherently, the fishery-dependent model is based on catch data from a time when there was a larger and more productive population of white abalone, predominantly found at San Clemente Island, Tanner Bank, and Cortes Bank. The fishery-independent model is based on presence data collected from fishery-independent surveys conducted throughout the SCB after the crash and closure of the fishery when the ocean was more impacted by climate change and the white abalone population was greatly reduced and lost its historical spatial structure.

These data nuances also contribute to the slight differences in rankings of importance and marginal effects of environmental factors. Both models generally described suitable habitat as relatively shallow (0–500 m) areas with intermediate lengths of coastline (10–100 km) and seabed slope (1–4°), and SST between 10 and 13°C. However, the fishery-dependent model exhibited a stricter distribution of suitable habitat than the fishery-independent model, displayed by fewer areas with habitat suitability ≥ 0.5. Furthermore, habitat suitability inferred from the fishery-dependent model appeared somewhat outdated, specifically with regards to SST since climate change has caused SST to rise since the time of the fishery and is projected to continue its upward trajectory [18, 19]. For that reason, the fishery-dependent model presents habitat suitability that may not be sensible in today’s warming climate, which is fairly evident in the lack of areas with habitat suitability ≥ 0.5 in the future projections. In contrast, the fishery-independent model reveals a broader distribution of suitable habitat (many areas with habitat suitability ≥ 0.5) resulting from observations made throughout the SCB when SSTs were slightly warmer. These conditions (broader distribution of suitable habitat and more realistic SST observations) render definitions of suitable habitat in the fishery-independent model to be more applicable to outplanting efforts, especially in the future across the state of California. The fishery-dependent model defines suitable habitat of the white abalone population when it was more abundant, suggesting it is a better representation of habitat suitability compared to the fishery-independent model based on observations from a remnant population. These respective strengths and drawbacks need to be considered when using this study to direct outplanting efforts.

This study has some limitations in defining suitable habitat for white abalone throughout the species’ native range due to the spatial resolution and spatial structure of the fishery-dependent data. The spatial resolution of the coarse-scale models is a considerably larger spatial extent than that encountered by an individual white abalone. This disagreement in spatial resolution consequently produced some differences in model outputs, particularly in the definitions of suitable habitat. For example, suitable habitat was characterized by habitat found in 0–500 m of water, which is a much larger range than the depth range of observed white abalone [2, 7, 13, 15]. This large range in selection on seabed depth is a direct artifact of aggregating data over such a large spatial extent. The zero-inflation and skewness exhibited in the fishery-dependent catch data made it challenging to explain variability and accurately model catch, even with a log transformation. This was especially apparent with the overprediction of zeros and underprediction of positive catch values. Future studies should explore the use of delta generalized linear models, which are more appropriate for working with zero-inflated and skewed data.

#### Fine-scale models

In the fine-scale models, seabed depth, VRM, and seabed slope had the greatest influence on habitat suitability for white abalone. While seabed rugosity and slope had the same effect on habitat suitability across all study areas, similar to what was observed in previous studies [2, 3], selection on depth differed between the offshore sites and the southern California coast. While this inconsistency in depth distribution between the offshore sites and the mainland may be an artifact of different depths surveyed using the ROV and SCUBA, it could also be an effect of light limitation and its consequential effects on kelp forest presence and growth. Terrestrial runoff and the resuspension of fine sediments reduces water clarity and light penetration along the mainland coast and restricts the distribution of kelp forests to ≥ 20 m. Coarser sediments and lower levels of runoff, as seen at the Channel Islands, result in relatively clear waters, allowing for kelp forests to extend to deeper depths [35].

Previous studies identified brown algae species to be a staple food source for white abalone [2, 15], suggesting that suitable habitat would be confined to areas of high kelp persistence. While we observed a positive relationship between habitat suitability and kelp persistence in waters surrounding San Diego, Palos Verdes exhibited the opposite relationship. This deviation in habitat suitability in relation to kelp persistence seen at Palos Verdes may be an artifact of settlement in suboptimal habitat. These individuals may have evaded fishing pressure because they were located in areas that either were perceived as less suitable by fishermen or had lower catch per unit effort, both of which may indicate suboptimal habitat. For Santa Barbara and Santa Catalina Islands, where the model included predator and competitor variables, habitat suitability was greatest in areas of low predator diversity and low competition (i.e., urchin abundance), consistent with past studies [15] and ecological theory of predator-prey and competitor dynamics. Interestingly, the model identified suitable habitat in all areas where two-spot octopus were present, irrespective of abundance level. This disparity between our model results and ecological theory may be due to a number of reasons, including settlement in poor habitat and size-dependent predator escapement. Hofmeister et al. [36] examined the behavior of SCB kelp forest predators in response to a concentrated increase in the abundance of juvenile red abalone (*H*. *rufescens*) during a restoration stocking experiment and discovered that octopus, specifically, will exploit the influx of prey. These findings recognize the high predation pressure octopus place on juvenile abalone immediately following outplanting. However, vulnerability to predation is highest during the early post-settlement life history stages, and decreases with size and age as individuals’ shells become stronger and larger and the animals are more able to adhere to the rocky substrate [3]. The presence of these adult individuals suggests an evasion of predation pressure given their size, and may not accurately represent suitable habitat for juvenile abalone.

The fine-scale models capitalize on the strengths of the fishery-independent datasets; the spatial accuracy of the species distribution data allowed suitability to be defined at a scale more likely to determine the presence and movement of white abalone. It is worth noting that environmental data, specifically biological variables (kelp persistence, temperature, salinity, etc.), had the coarsest resolution and limited the spatial resolution of the fine-scale models. While a majority of species observations had a spatial accuracy of ± 1 m, biological data does not exist at this spatial scale in the areas of interest, limiting our modeling efforts and performance. Nonetheless, the better understanding of factors that determine habitat suitability from the fine-scale models provides valuable insight for the selection of sites for experimental outplanting efforts and the refinement of sampling designs for surveys that monitor remnant populations at these sites.

### Conservation implications and next steps

Both coarse-scale and fine-scale analyses of habitat suitability using fishery-dependent and fishery-independent data provide information that can improve the efficiency and success of ongoing and future outplanting efforts. We suggest an experimental approach for outplanting that allows for recovery while also collecting requisite data at appropriate scales to advance our understanding of what constitutes suitable habitat for white abalone habitat in a changing environment. Areas of high suitability like San Clemente Island, based on the fishery-dependent model, and the Northern Channel Islands (e.g., San Miguel Island) and particular areas along the mainland coast of southern California (San Diego and Palos Verdes), based on the fishery-independent model, propose potential habitat capable of supporting adult white abalone populations. While these modeling results may be instructive, managers must weigh these outcomes with other associated variables, including proximity to hatchery, ease of access, etc.

At potential outplanting locations, fine-scale model results, particularly the marginal effects of influential environmental variables, can inform outplanting experiments with variable gradients to test across (e.g. depth ranges of 30–70 m). Given sufficient numbers of captive-bred juveniles, the experimental design can assume a randomized block design to test all possible habitat combinations and enhance understanding on how these habitat characteristics translate to juvenile survival and growth. Furthermore, the collection of environmental and biological data at appropriate scales that we were unable to include efficiently in our analyses (e.g., temperature, dissolved oxygen, pH) will allow for additional analyses of white abalone habitat suitability. The first release of captive-bred juvenile white abalone occurred in the fall of 2019 in two areas included in this study. Fine-scale model outputs for these respective areas and suggested experimental methods can be directly applied to future efforts to maximize the likelihood of survival and the establishment of a self-sustaining wild population.

Although SST was among the least important variables in both the coarse-scale fishery-dependent or fishery-independent model, temperature has been shown to influence the survival [15, 20], growth [15], reproduction [37], and disease risk [38] of abalone, in general. Additionally, temperature affects the health and persistence of giant kelp (*Macrocystis pyrifera*), a common food source for white abalone in the SCB. For example, long periods of warmer temperatures observed during El Niño events resulted in widespread declines of giant kelp and the deterioration of subsurface canopies [38]. The SCB is experiencing an increase in SST [39]. If temperatures continue to rise, as is expected under climate projections, kelp forests will likely decline and negatively affect the growth [15], survival [15, 20], and reproduction [37] of wild and outplanted abalone populations. Given the potential influence of climate change on outplanting activities in the SCB, outplanting efforts may benefit from investigations into areas expected to experience delayed or reduced increases in SST, such as north of Point Conception. While SST is expected to increase in northern California into the future, it will remain within a suitable range for survival, growth, reproduction, and disease resistance for white abalone. Despite the relative lack of suitable habitat located in these more northern areas based on the fishery-dependent model projections, the fishery-independent future model projections identified areas in the north and outside of the native range of white abalone to have suitable habitat, specifically Cape Mendocino, Point Arena, Point Reyes, and Monterey. Given their higher latitude, these areas may experience fewer effects of climate change further into the future, under worsening RCP scenarios, compared to those in the SCB and provide additional suitable habitat capable of supporting the long-term recovery of a white abalone population as the environment changes. Introducing a species outside of its native range can impact the designated outplanting habitat and its native species. These new potential interactions should be considered and measured throughout the outplanting experiment.

By modeling the distribution of white abalone, we identified suites of environmental characteristics that described relative suitable habitat over different time periods and spatial scales, and use this information to identify locations that recovery efforts could focus on. Future model projections suggest the possibility of several locations in northern California, outside the native range of white abalone, to support the recovery of this endangered species in a changing environment. The findings from our fine-scale analysis can be used to inform an experimental outplanting framework intended to improve our understanding of the factors that maximize the survival of captive bred individuals at outplanting locations. Much work has been done to protect, monitor, and artificially enhance the remnant wild populations, and the data from these efforts made this analysis possible. A statistically rigorous experimental outplanting program with better environmental monitoring would help improve upon these efforts.

## Supporting information

S1 File Definitions of environmental variables, methods of derivation, and Maxent model evaluation. (DOCX)

S2 File Additional figures for coarse-scale modeling results. (DOCX)

S3 File Additional figures for fine-scale modeling results. (DOCX)
