# Supplementary material for: Modeling the past, present, and future distributions of endangered white abalone (Haliotis sorenseni) to inform recovery efforts in California
Source: PLoS One. 2021 Nov 17;16(11):e0259716. doi: 10.1371/journal.pone.0259716 (PMC8598040; doi:10.1371/journal.pone.0259716)
Supplement: S1 File — (DOCX) [file pone.0259716.s001.docx]

**Appendix S1: Definitions of Environmental Variables, Methods of Derivation** **and Maxent Model Parameter Evaluation**

**Depth**

Depth represents the ocean’s depth, in meters, relative to sea level. As depth increases, depth values become more negative. We obtained coarse-scale [1] and fine-scale [2] depth data at spatial resolutions of 200 x 200 m and 2 x 2 m, respectively. Depth values range from 0 to -4,000 m and 0 to -200 m for coarse-scale and fine-scale data, respectively. In ArcGIS we aggregated both coarse-scale and fine-scale data to their respective spatial resolutions for modeling using the Aggregate Tool.

**Vector Ruggedness Measure (VRM)**

VRM measures the terrain roughness of the seafloor as the variation of grid cells in three-dimensional space within a predefined neighborhood. VRM values can range from 0 (flat, no terrain variation) to 1 (rough, complete terrain variation), while typical seafloor terrain ranges from 0 to about 0.4 [3].

We obtained available fine-scale VRM [2] at a spatial resolution of 2 x 2 m. For areas with missing data, we derived VRM from respective bathymetry data using ArcGIS extension Benthic Terrain Modeler (BTM) with a neighborhood size of 3 m [4]. The methods executed to derive VRM from bathymetry data were comparable with methods taken by researchers at California State University, Seafloor Mapping Lab (CSUMB SFML) [2]. In ArcGIS we aggregated VRM data to a spatial resolution of 10 m^2^ using the Aggregate Tool.

**Slope**

Slope is the incline or steepness of the seafloor and can be measured in degrees from a horizontal plain. Slope values are larger when an area of relief has a greater degree of slope. At a coarse-scale, areas of greater slope can be representative of continental shelves, while at a fine-scale areas of greater slope may represent rock structures or ledges.

We derived coarse-scale slope data from respective bathymetry data using ArcGIS extension BTM. When available, we obtained fine-scale slope data [2] at a spatial resolution of 2 x 2 m. For areas with missing data, we derived slope from bathymetry data using ArcGIS extension BTM. The methods to derive slope from bathymetry data were comparable to methods executed by researchers at CSUMB SFML [2]. Slope values range from 0 to 6 degrees and 0 to 60 degrees for coarse-scale and fine-scale data, respectively.

**Substrate**

Substrate is a classification of roughness (soft bottom [0] and rough bottom [1]), but can serve as a proxy for seafloor type: sand/sediment (0) and rock (1). We obtained available fine-scale substrate data [2] at a spatial resolution of 2 x 2 m. For areas where data is lacking, we derived substrate data from the interpretation of VRM data, using an appointed threshold to differentiate soft from rough substrate. VRM values equal to or above the selected threshold were categorized as “rough” while VRM values below were categorized as “smooth.” We selected site specific thresholds based on accuracy assessments that compared “supervised” classifications with visual classifications. Supervised classifications were based on interpretation of VRM values relative to a standardized set of appointed thresholds while visual classifications were based on visual interpretation of substrate from hillshade and shaded relief. We elected thresholds that minimized and balanced Type 1 and Type 2 errors amongst 100 randomly selected points for each site to be used to classify substrate. The methods executed to derive substrate from VRM data were comparable to methods executed by researchers at SFML CSUMB [2]. In ArcGIS we combined data into study areas using the Combine Tool and aggregated data to a spatial resolution of 10 using the Aggregate Tool.

**Kelp Persistence Index**

We defined kelp persistence as a measure of the binary maximum likelihood of *Macrocystis pyrifera* presence through time. Kelp persistence values range from 0 to 1, where small values represent low persistence and large values represent high persistence. Because kelp experience reduced or negative growth months in the summer, we focused our analysis on spring months (April through June), a period when cool, nutrient-rich bottom waters are brought to the surface through upwelling, thus providing an ideal environment for growth and an appropriate indicator of kelp presence.

We obtained data for the coastal areas of southern California, including northern and southern Channel Islands and banks that overlapped with fine-scale study areas from 1993 through 2017 [5]. We converted bi-monthly satellite wet biomass estimates at a spatial resolution of 30 m^2^ to a binary classification of presence where if wet biomass was greater than 0 it was considered present and if wet biomass was 0 it was considered absent. If kelp was observed present at least once during these 3 months, kelp was considered present for that specific year. We calculated the binary maximum likelihood for each pixel by dividing the number of years kelp was present by the total number of years observed. In ArcGIS we used inverse distance weighting (IDW) spatial interpolation to develop kelp persistence estimates at a finer resolution (10 m^2^) assuming spatially distributed objects are spatially correlated. Each measured point has a local influence that diminishes with distance, giving greater weights to points closest to the prediction location and diminishing weight as a function of distance. We justified the use of spatial interpolation by assuming that kelp presence did not vary dramatically within 30 m^2^.

For IDW settings, we set the search radius to 3 nearest input sample points to be used to perform interpolation and kept the power at the default value of 2. Power controls the significance of surrounding points on the interpolated value. A higher power results in less influence from distant points. It can be any real number greater than 0, but the most reasonable results will be obtained using values from 0.5 to 3. The default is 2.

**Temperature and Salinity**

Temperature and salinity are measured in °C and practical salinity unit (psu) respectively. We retrieved surface temperature and salinity data for time periods associated to fishery-dependent (1959-1991) and fishery-independent (1993-2017) white abalone occurrence data [6]. For both time periods, we averaged measurements over years at each sample site. In ArcGIS, we used IDW spatial interpolation to develop sea surface temperature and salinity estimates at spatial resolution of approximately 20 km^2^.

**Predator Diversity**

Predator diversity is the average proportion of abalone predators present through time. Among the suite of known predator species, we included *Octopus* spp. (*Octopus bimaculatus* and *Octopus rubescens*), *Panulirus interruptus*, *Semicossyphus pulcher*, *Scorpaenichthys marmoratus, Myliobatis californica,* and *Heterodontus francisci* in the analysis. Predator diversity values range from 0 (no predators present) to 1 (high predator presence and diversity).

We acquired predator data [7] for southern California from 1994 to 2017. In R, we converted species’ abundance data for each survey to a binary classification of presence where if abundance was greater than 0 it was considered present (1) and if species were not detected in a survey it was considered absent (0).  We derived predator diversity by first calculating the sighting frequency for each predator at each dive location (Equation 1) and then calculating the mean proportion of predators present for each dive site (Equation 2). Dive locations that included at least five conducted surveys were used in analysis to increase confidence in analysis results. Using the Buffer Tool in ArcGIS, we developed buffers around survey sites (600 meters in radius) that assumed its corresponding predator diversity values to mimic the search area of survey divers. In areas where buffers overlapped, the highest predator diversity value was adopted.

Equation 1. Sighting Frequency

$${SF}_{p}=\frac{n_{p}}{n_{s}}$$

Where *n_p_* is the number of surveys where a predator species, *p,* was observed and n_s_ is the number of surveys conducted at location s.

Equation 2. Mean Proportion of Predators Present (Predator Diversity)

$$\frac{{SF}_{1}+{SF}_{2}+{SF}_{3}\ldots+{SF}_{p}}{N_{n}}$$

Where ${SF}_{p}$ is the sighting frequency for each predator species, and $N_{n}$ is the number of predator species (in this case 7 species).

**Two Spot Octopus and Urchin (Purple and Red) Abundance Scores**

Abundance score is an estimate of abundance for a species that accounts for non-sightings. Values range from 0 (no abundance) to 4 (high abundance) for California two-spot octopus and 0 to 8 for urchin (because it represents a collective measure for red and purple urchin). We calculated abundance scores (Equation 4) from species’ density scores (Equation 3), derived from Reef Environmental Education Foundation’s [7] abundance category data and sighting frequency for each dive location. Density score is a weighted average index based on the frequency of observations in different abundance categories. Dive locations that included at least five conducted surveys were used in analysis to increase confidence in analysis results.  We conducted similar methods in ArcGIS to those conducted for predator diversity analysis.

Equation 3. Density Score

$$D=\frac{\left( nS\times1 \right)+\left( nF\times2 \right)+\left( nM\times3 \right)+(nA\times4)}{nS+nF+nM+nA}$$

where $nS$, $nF$, $nM$, and $nA$ represented the number of times each abundance category (Single, Few, Many, Abundant) was assigned for a given species.

Equation 4. Abundance Score

$$A=D\times{SF}_{p}$$

**Length of Coastline**

Length of coastline is defined as the distance in km of coastline within each CDFW statistical fishing block and ranges from 0 to 150 km. We obtained a coastline shapefile of California (including the Channel Islands) [1] and derived the total length of coastline per CDFW fishing block in ArcGIS using the Measure tool.

**Preliminary Correlation Diagnostics**

Of the collected and derived coarse-scale environmental variables, we selected variables with a Pearson Correlation Coefficient (PCC) <0.7 as input into the model. When a pair of environmental variables had a PCC >0.7, we omitted the variable (sea surface salinity) with less established knowledge on its relationship to white abalone habitat preference and survival. This filtering resulted in four variables selected as model inputs: seafloor depth (m), seafloor slope (degrees), length of coastline (km), and mean SST (degrees C). Of the collected and derived fine-scale environmental variables, we selected variables with a PCC < 0.7 to be incorporated in fine-scale analysis. When a pair of environmental variables had a PCC >0.7, we omitted the less informative variable that could be represented in the correlated variable (substrate), resulting in seven variables included in the model: depth (m), slope (degrees), VRM, kelp persistence index, predator diversity index, California two-spot octopus abundance index, and urchin abundance index. We were unable to include oceanographic environmental variables due to limitations in spatial and temporal coverage and resolution of available data.

**Maxent Model Parameter Evaluation**

We tested a range of regularization levels, varying from 1 to 10 and feature types (linear and quadratic), and conducted model selection across parameterization options using AICc for each study area. Most of the study areas denoted the best performing model to include a regularizing multiplier of 1 and linear feature type, and were thus used in modeling across all study areas (Figure 1).

Figure 1. Evaluation for *Haliotis sorenseni* resulting from Maxent models made across varying feature classes (linear and quadratic) and regularization multipliers (1-10) at the different study areas. Each panel visualizes corresponding model AICc values for San Clemente Island (a), Santa Catalina and Santa Barbara Islands (b), Tanner and Cortes Banks (c), San Diego (d), and Palos Verdes (e), Southern California Bight (f).


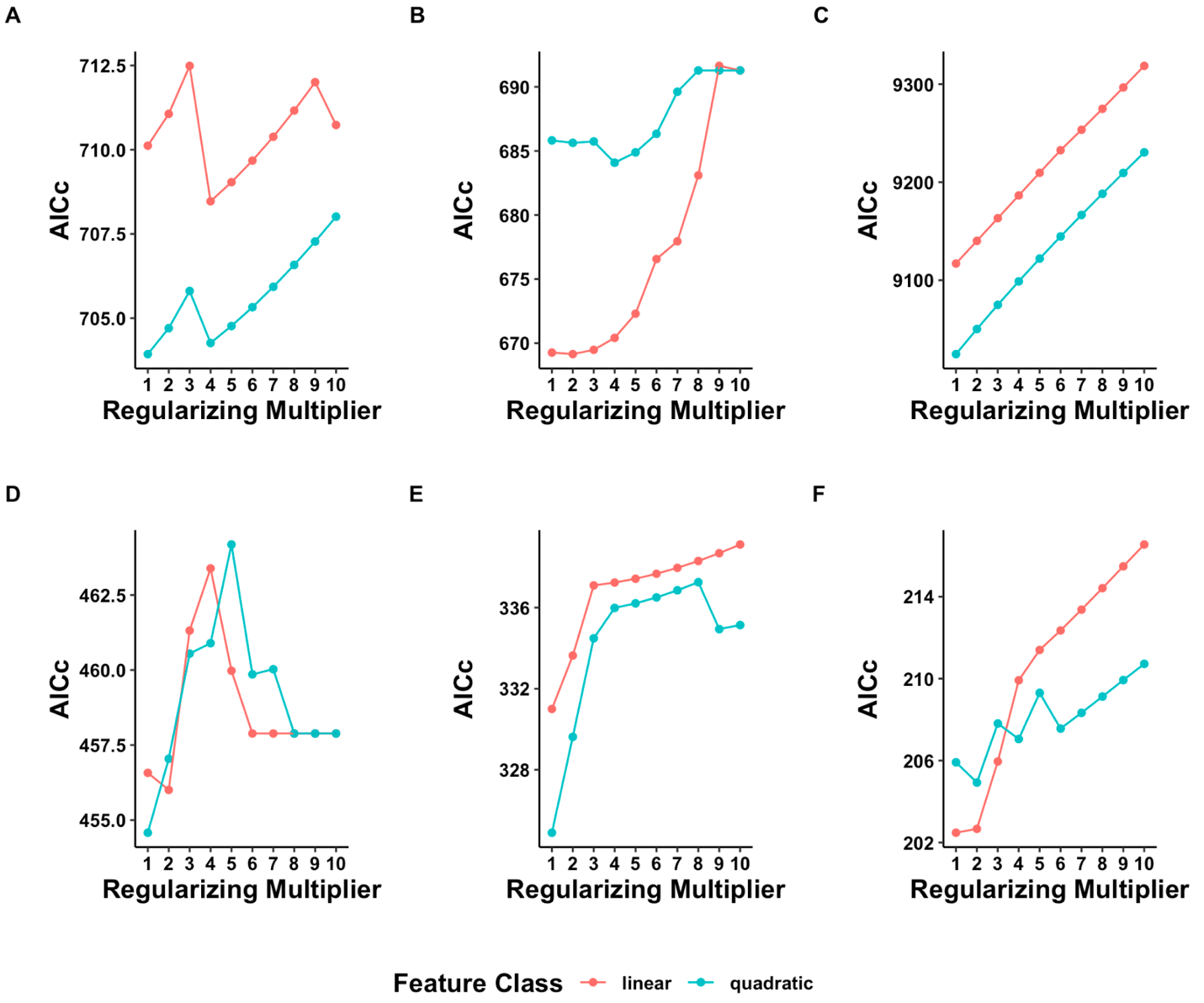


**References**

1. Teal K, Richardson A, Wong-Coppin V. Bathymetry Project (Second Edition). 2002. California Department of Fish and Game, Monterey, California. Available from https://www.wildlife.ca.gov/Conservation/Marine/GIS/Downloads (accessed June 2017).
2. CSUMB SFML. SFML Data; 2008. CSUMB SFML.
3. Sappington JM, Longshore KM, Thompson, DB. Quantifying landscape ruggedness for animal habitat analysis: a case study using bighorn sheep in the Mojave Desert. The Journal of wildlife management; 71(5), 1419-1426. https://seafloor.otterlabs.org/SFMLwebDATA.htm.
4. Walbridge, S, Slocum N, Pobuda M, Wright DJ. Unified geomorphological analysis workflows with Benthic Terrain Modeler. Geosciences. 2018; 8(3), 94.
5. Bell T, Cavanaugh K, Siegel D. SBC LTER: Time series of quarterly NetCDF files of kelp biomass in the canopy from Landsat 5, 7 and 8, since 1984 (ongoing) ver 13; 2020 Environmental Data Initiative.
6. NOAA SWFSC. CalCOFI NOAAHydros. 2017. ERDDAP. https://coastwatch.pfeg.noaa.gov/erddap/tabledap/erdNOAAhydros.graph
7. REEF. 2007. Reef Environmental Education Foundation Volunteer Fish Survey Project Database. World Wide Web electronic publication. https://www.reef.org (accessed June 2017).
