# Supplementary material for: Modeling the past, present, and future distributions of endangered white abalone (Haliotis sorenseni) to inform recovery efforts in California
Source: PLoS One. 2021 Nov 17;16(11):e0259716. doi: 10.1371/journal.pone.0259716 (PMC8598040; doi:10.1371/journal.pone.0259716)
Supplement: S2 File — (DOCX) [file pone.0259716.s002.docx]

**Appendix S2: Coarse-scale Model Results**


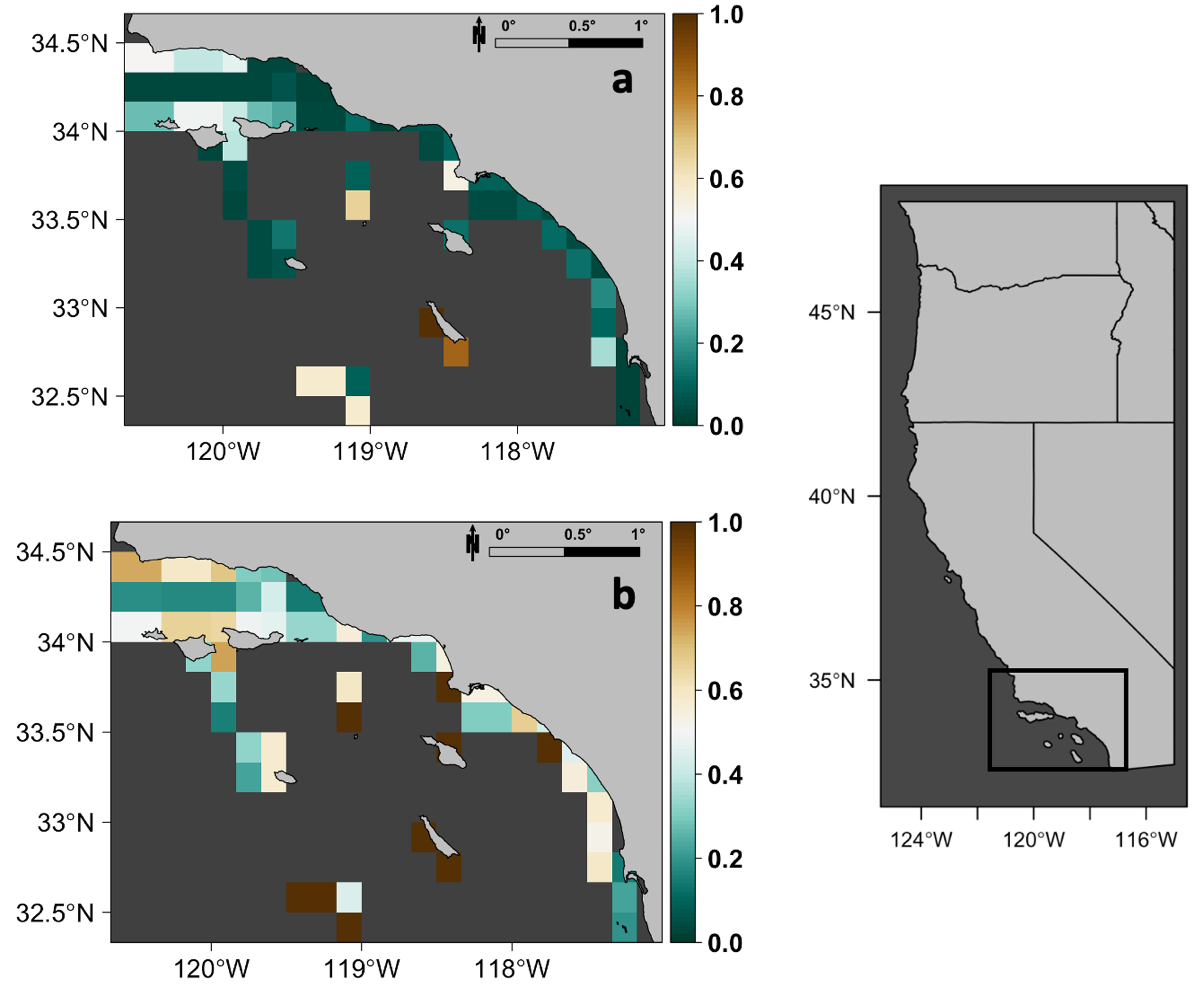


Figure 1. Predicted relative mean habitat suitability (a) and error in predictions (b) based on Random Forest and historical, fishery-dependent data. The relative mean habitat suitability is averaged over 10000 trees. Error in predicted relative habitat suitability is represented as the 95% quantile range of predictions. The color scale to the right of the panels delineates mean habitat suitability or variability in predictions of relative habitat suitability depending on the panel. Predicted relative mean habitat suitability and error in predictions is visualized for fishing blocks ≤ 500 m in depth. The reference map depicts the west coast of the United States with the study area identified by the solid bounding box.


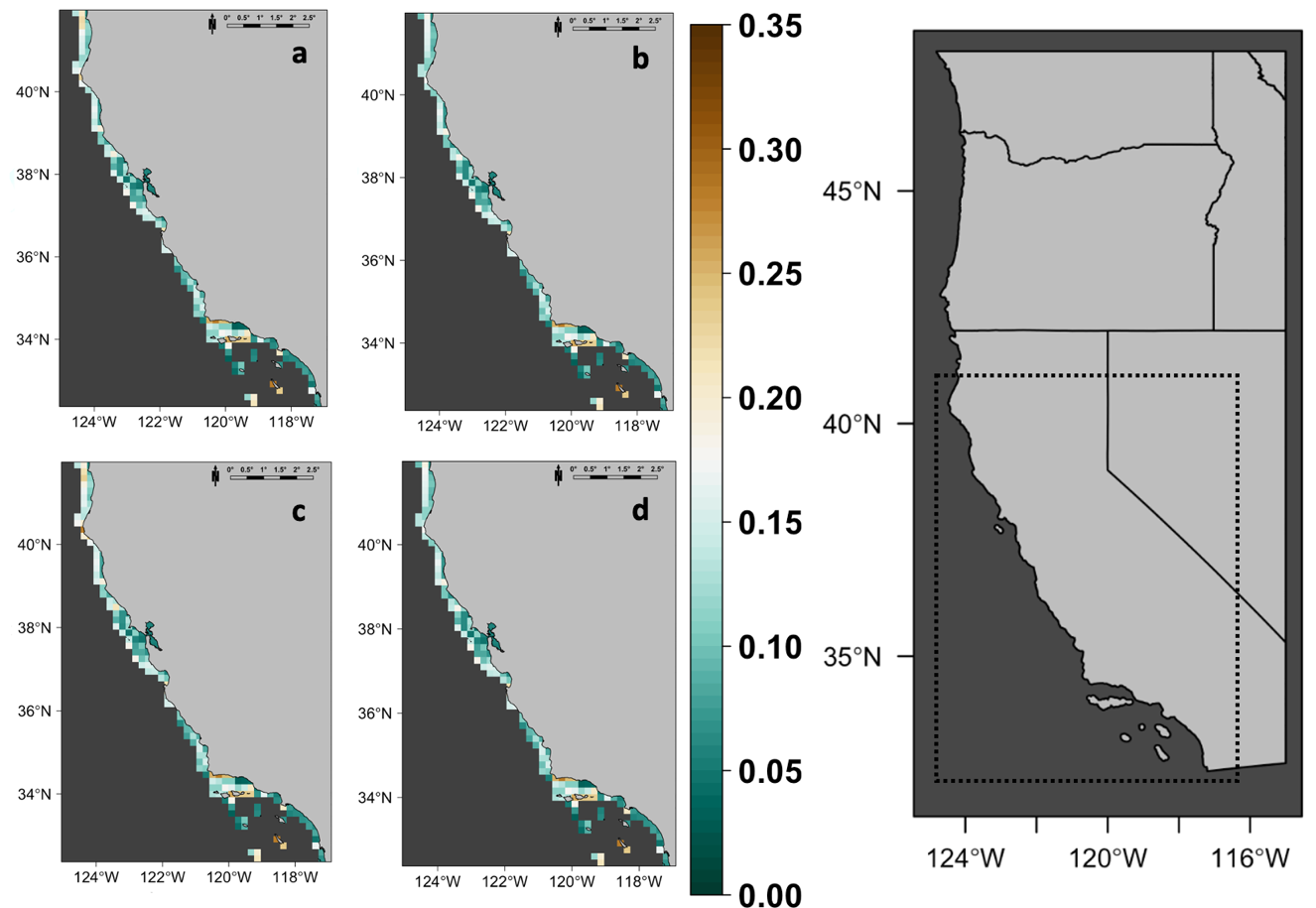


Figure 2. Predicted relative mean habitat suitability in California waters for 2050 based on Random Forest and historical fishery-dependent data under RCP 2.6 (a), RCP 4.5 (b), RCP 6.0 (c), and RCP 8.5 (d) scenarios. Each map represents projected relative mean habitat suitability averaged over 10000 trees. The color scale to the right of the panels delineates relative habitat suitability, ranging from unsuitable (0.0) to most suitable (0.35). Predicted relative mean habitat suitability is visualized for fishing blocks ≤ 500 m in depth. The reference map depicts the west coast of the United States with the modeling area identified by the dotted bounding box.


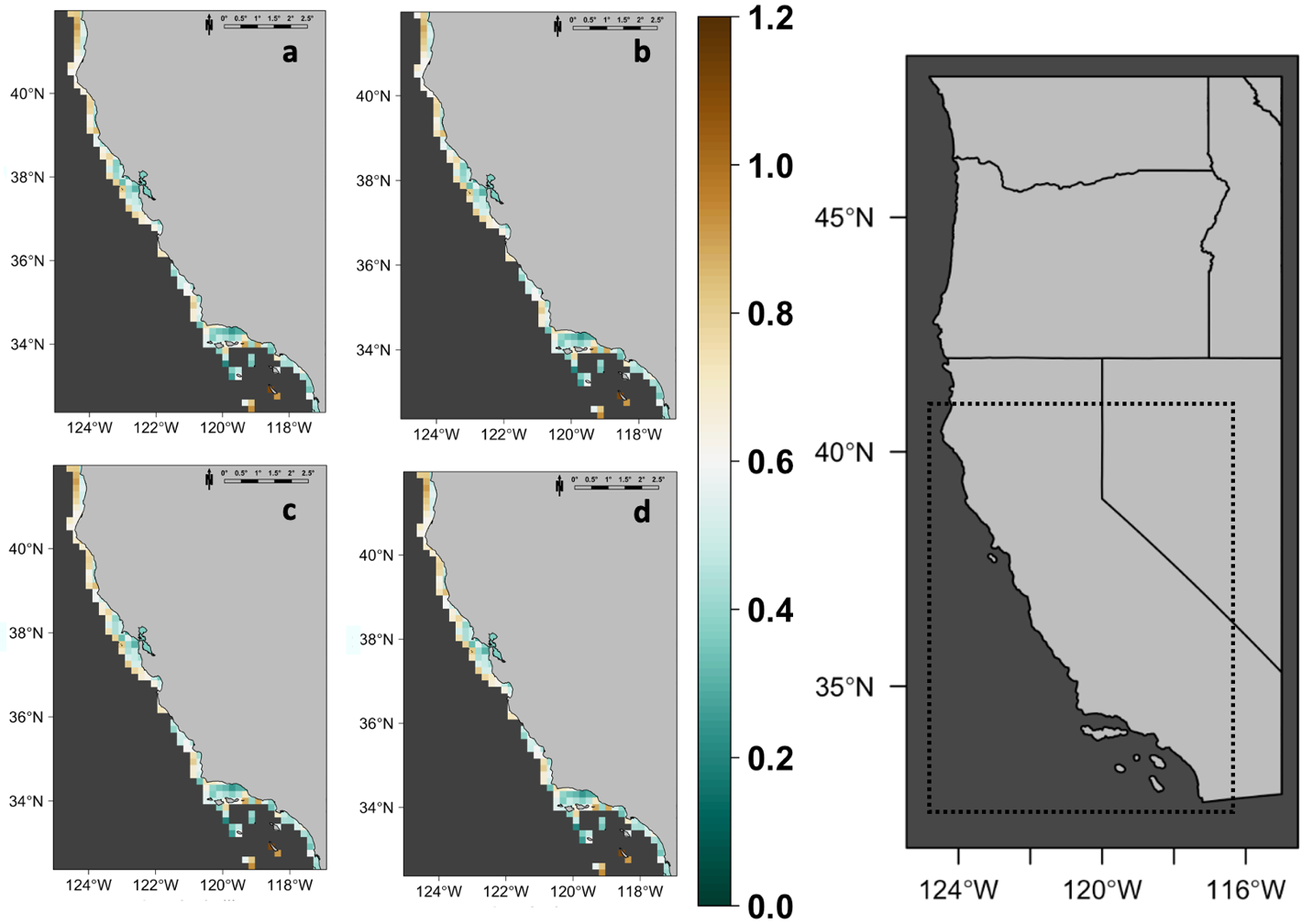


Figure 3. Error in projections for 2050 based on Random Forest and historical, fishery-dependent data under RCP 2.6 (a), RCP 4.6 (b), RCP 6.0 (c), and RCP 8.5 (d) scenarios. Each map represents the 95% quantile range of predictions of relative habitat suitability. The color scale to the right of the panels delineates variability in predictions of relative habitat suitability, ranging from low variability (0.0) to high variability (1.1). Error in predictions is visualized for fishing blocks ≤ 500 m in depth. The reference map depicts the west coast of the United States with the modeling area identified by the dotted bounding box.


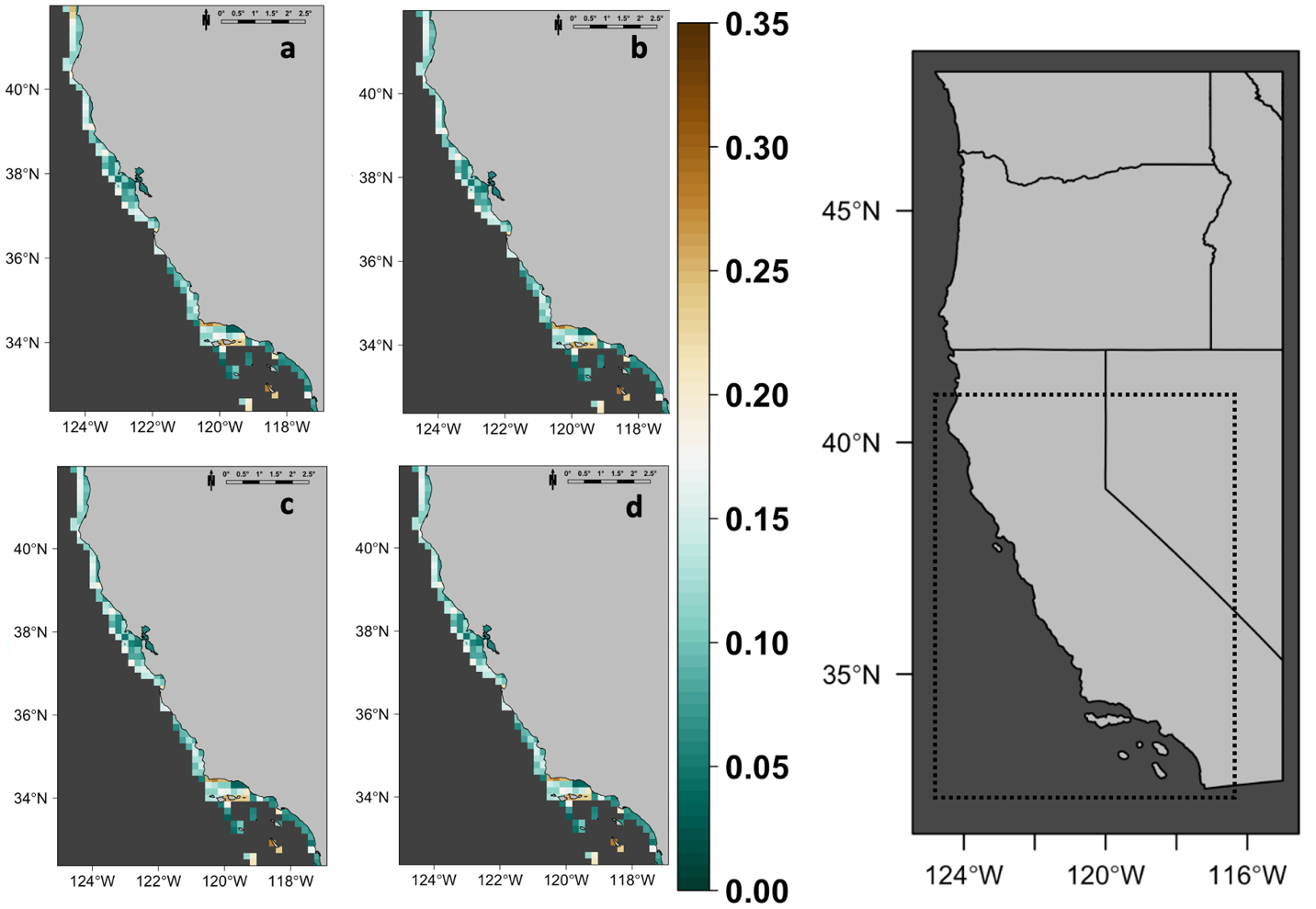


Figure 4. Predicted relative mean habitat suitability in California waters for 2100 based on Random Forest and historical fishery-dependent data under RCP 2.6 (a), RCP 4.5 (b), RCP 6.0 (c), and RCP8.5 (d) scenarios. Each map represents projected relative mean habitat suitability averaged over 10000 trees. The color scale to the right of the panels delineates relative habitat suitability, ranging from unsuitable (0.0) to most suitable (0.35). Predicted relative mean habitat suitability is visualized for fishing blocks ≤ 500 m in depth. The reference map depicts the west coast of the United States with the modeling area identified by the dotted bounding box.


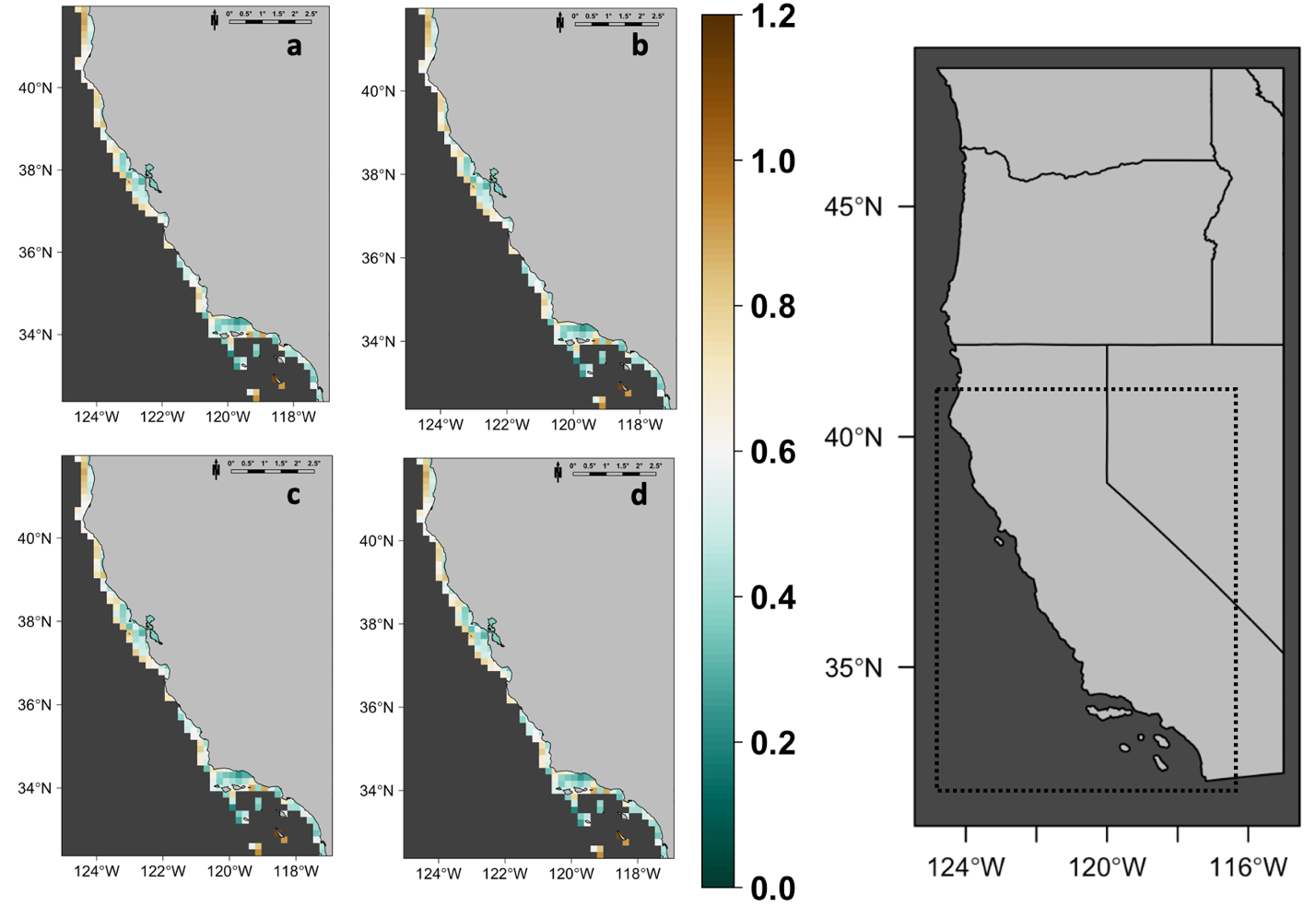


Figure 5. Error in projections for 2100 based on Random Forest and historical, fishery-dependent data under RCP 2.6 (a), RCP 4.6 (b), RCP 6.0 (c), and RCP 8.5 (d) scenarios. Each map represents the 95% quantile range of predictions of relative habitat suitability. The color scale to the right of the panels delineates variability in predictions of relative habitat suitability, ranging from low variability (0.0) to high variability (1.1). Error in predictions is visualized for fishing blocks ≤ 500 m in depth. The reference map depicts the west coast of the United States with the modeling area identified by the dotted bounding box.


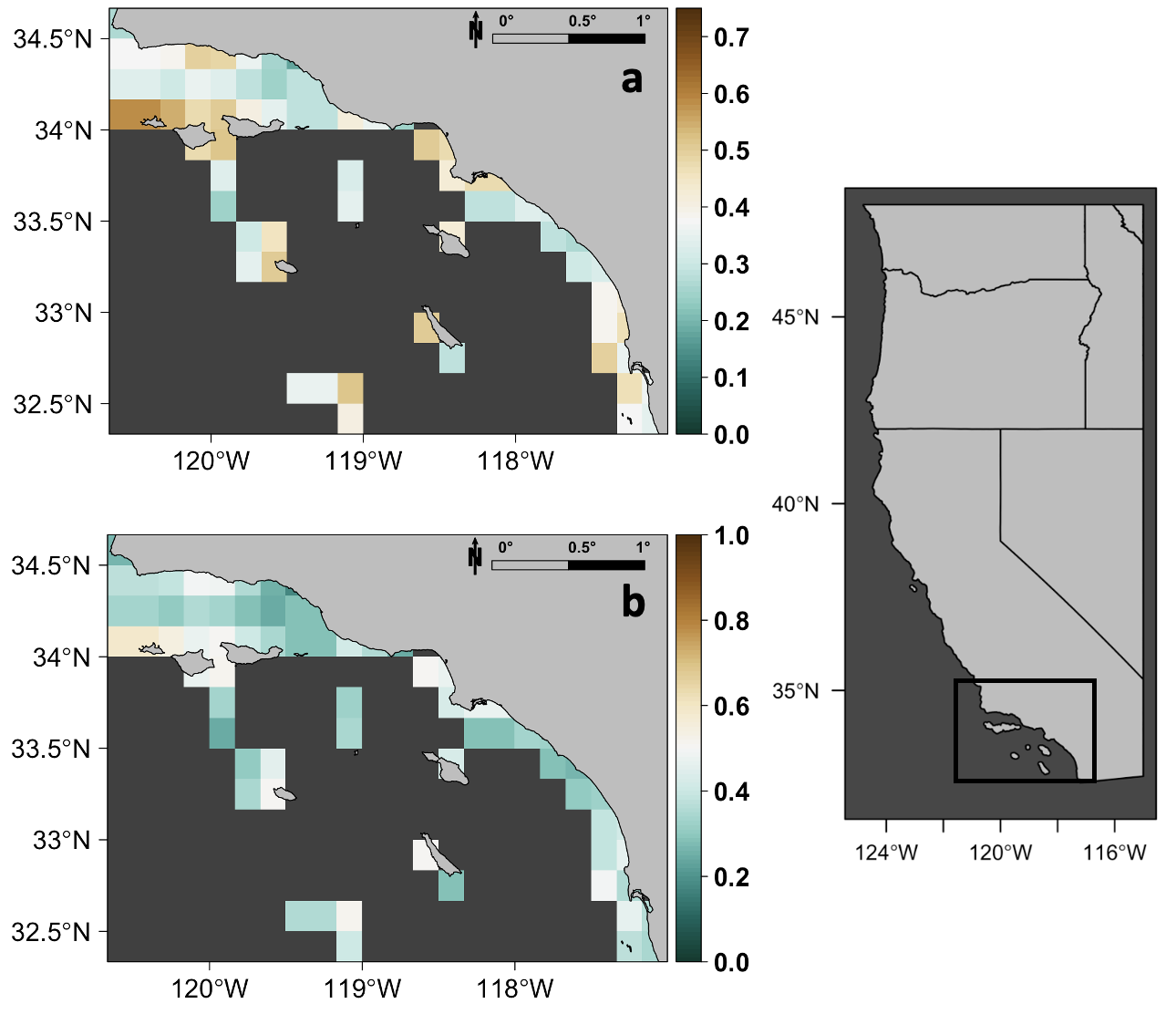


Figure 6. Predicted relative mean habitat suitability (a) and error in predictions (b) based on MaxEnt and contemporary, fishery-independent data. The relative mean habitat suitability is averaged over 100 model runs. Error in predicted relative habitat suitability is represented as the 95% quantile range of predictions. The color scale to the right of the panels delineates relative mean habitat suitability or variability in predictions of relative habitat suitability depending on the panel. Predicted relative mean habitat suitability and error in predictions is visualized for fishing blocks ≤ 500 m in depth. The reference map depicts the west coast of the United States with the study area identified by the solid bounding box.


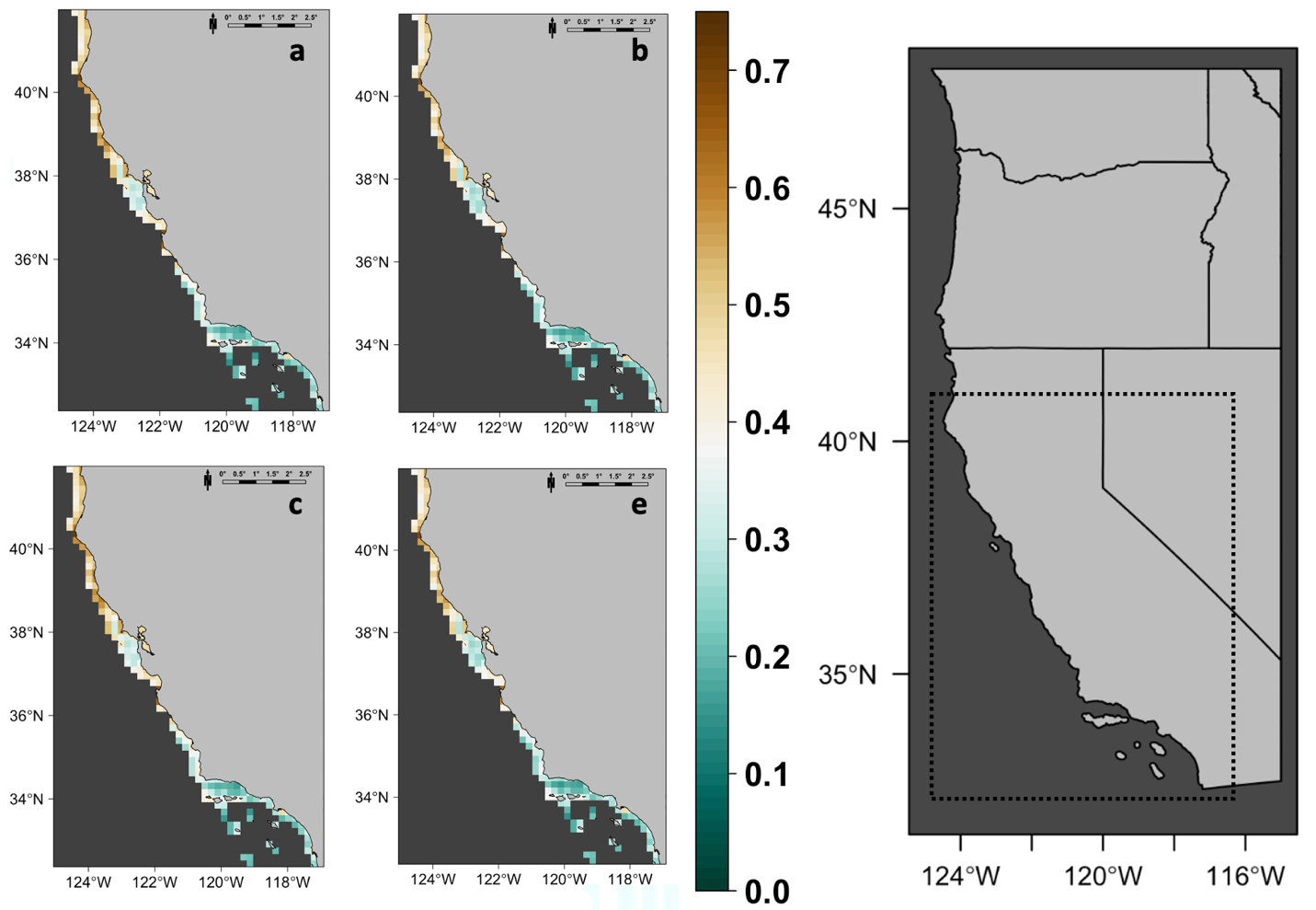


Figure 7. Predicted relative mean habitat suitability in California waters for 2100 based on MaxEnt and contemporary, fishery-independent data under RCP 2.6 (a), RCP 4.5 (b), RCP 6.0 (c), and RCP8.5 (d) scenarios. Each map represents projected relative mean habitat suitability averaged over 100 model runs. The color scale to the right of the panels delineates relative habitat suitability, ranging from unsuitable (0.0) to most suitable (0.7). Predicted relative mean habitat suitability is visualized for fishing blocks ≤ 500 m in depth. The reference map depicts the west coast of the United States with the modeling area identified by the dotted bounding box.


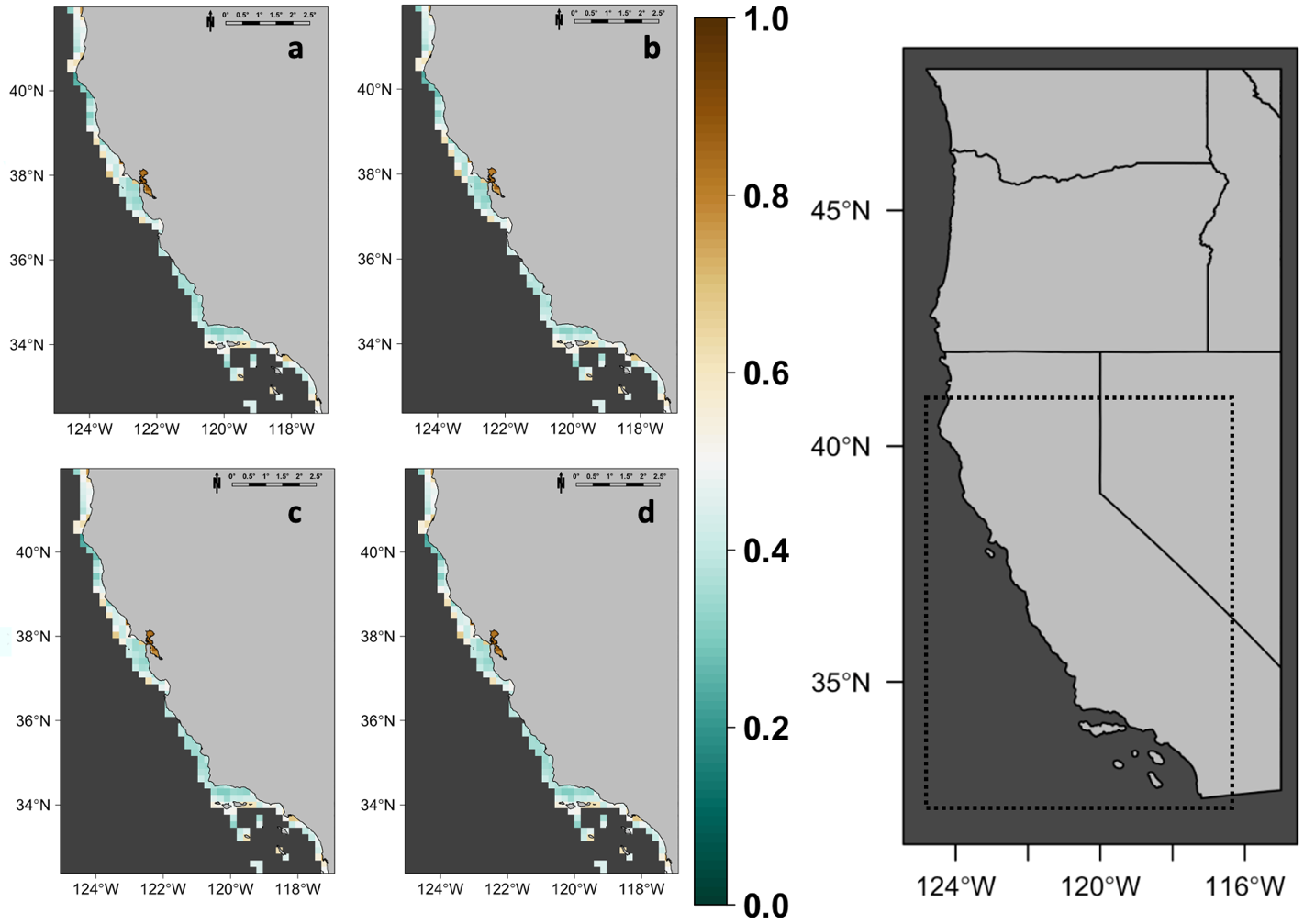


Figure 8. Error in projections for 2050 based on MaxEnt and contemporary, fishery-independent data under RCP 2.6 (a), RCP 4.6 (b), RCP 6.0 (c), and RCP 8.5 (d) scenarios. Each map represents the 95% quantile range of predictions of relative habitat suitability. The color scale to the right of the panels delineates variability in predictions of relative habitat suitability, ranging from low variability (0.0) to high variability (1.0). Error in predictions is visualized for fishing blocks ≤ 500 m in depth. The reference map depicts the west coast of the United States with the modeling area identified by the dotted bounding box.


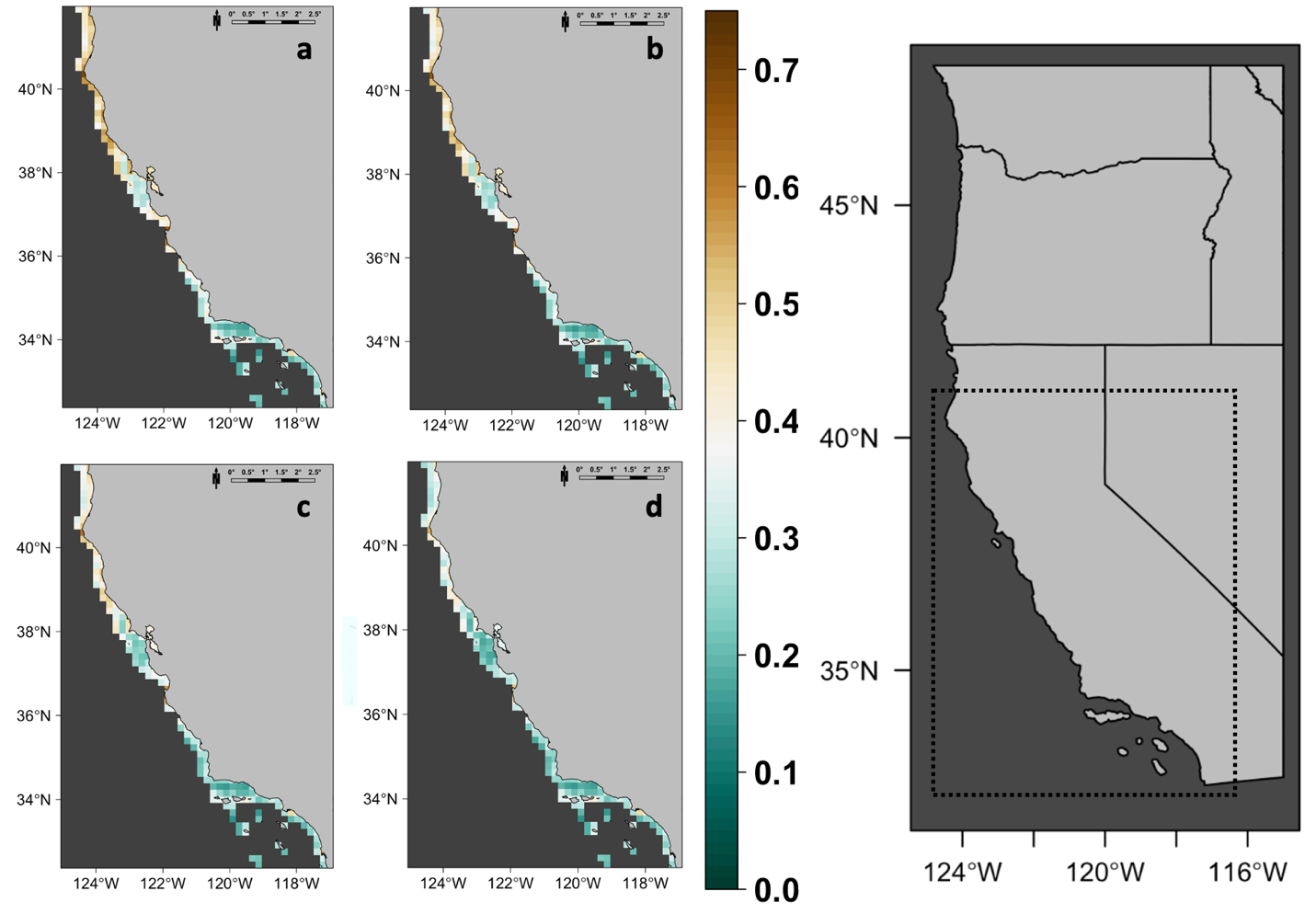


Figure 9. Predicted relative mean habitat suitability in California waters for 2100 based on MaxEnt and contemporary, fishery-independent data under RCP 2.6 (a), RCP 4.5 (b), RCP 6.0 (c), and RCP8.5 (d) scenarios. Each map represents projected relative mean habitat suitability averaged over 100 model runs. The color scale to the right of the panels delineates relative habitat suitability, ranging from unsuitable (0.0) to most suitable (1.0). Predicted relative mean habitat suitability is visualized for fishing blocks ≤ 500 m in depth. The reference map depicts the west coast of the United States with the modeling area identified by the dotted bounding box.


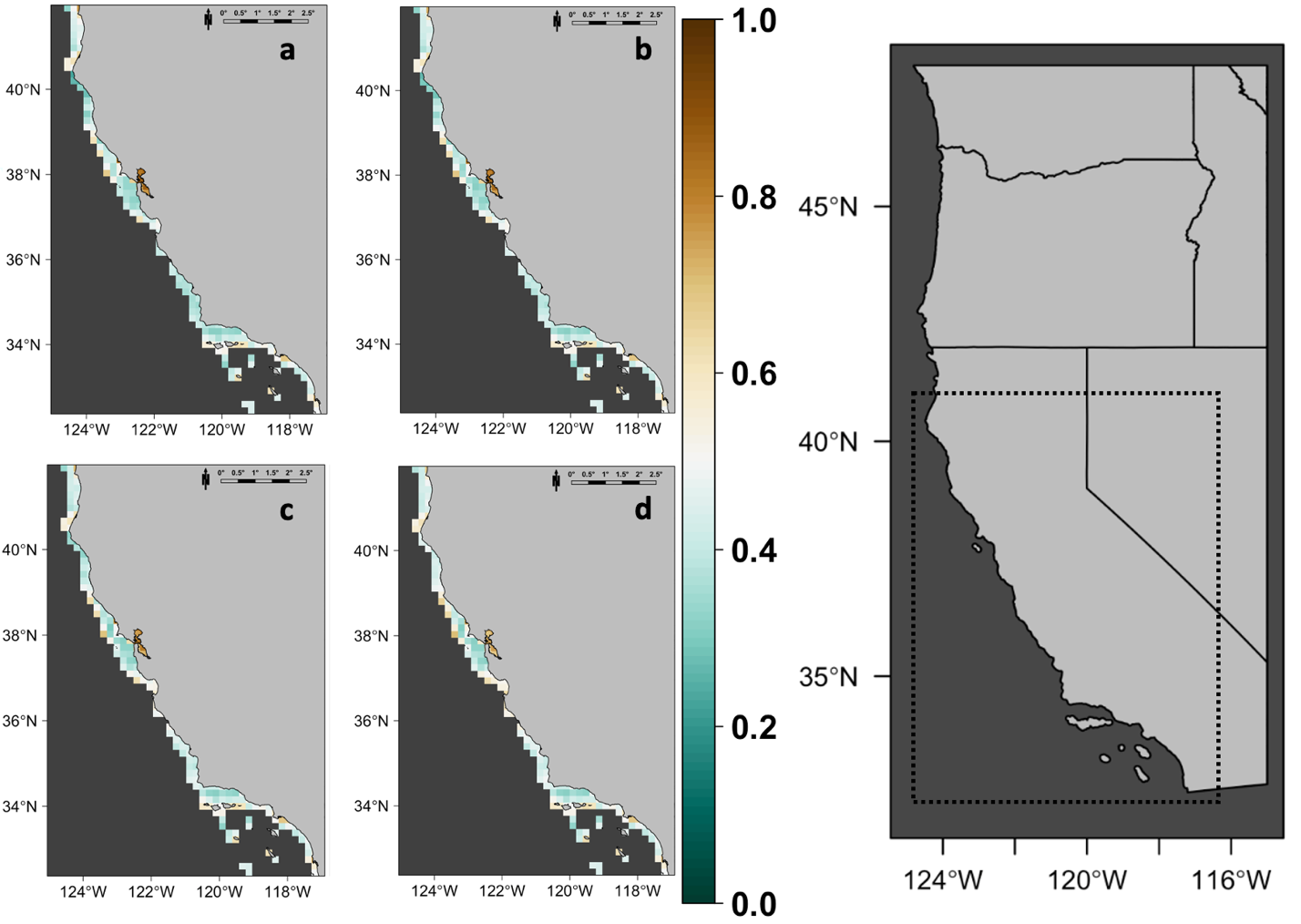


Figure 10. Error in projections for 2100 based on MaxEnt and contemporary, fishery-independent data under RCP 2.6 (a), RCP 4.6 (b), RCP 6.0 (c), and RCP 8.5 (d) scenarios. Each map represents the 95% quantile range of predictions of relative habitat suitability. The color scale to the right of the panels delineates variability in predictions of relative habitat suitability, ranging from low variability (0.0) to high variability (1.0). Error in predictions is visualized for fishing blocks ≤ 500 m in depth. The reference map depicts the west coast of the United States with the modeling area identified by the dotted bounding box.

Figure 11. Response curves for Depth (a), Slope (b), and VRM (c) developed by Random Forest using historical, fishery-dependent data. The black line represents the mean response of 100 model runs and the grey shaded region represents one SD of the mean.

Figure 12. Response curves for Depth (a), Slope (b), and VRM (c) developed by MaxEnt using contemporary, fishery-independent data. The black line represents the mean response of 100 model runs and the grey shaded region represents one SD of the mean.
