# Supplementary material for: Modeling the past, present, and future distributions of endangered white abalone (Haliotis sorenseni) to inform recovery efforts in California
Source: PLoS One. 2021 Nov 17;16(11):e0259716. doi: 10.1371/journal.pone.0259716 (PMC8598040; doi:10.1371/journal.pone.0259716)
Supplement: S3 File — (DOCX) [file pone.0259716.s003.docx]

**Appendix S3: Fine-scale Model Results**


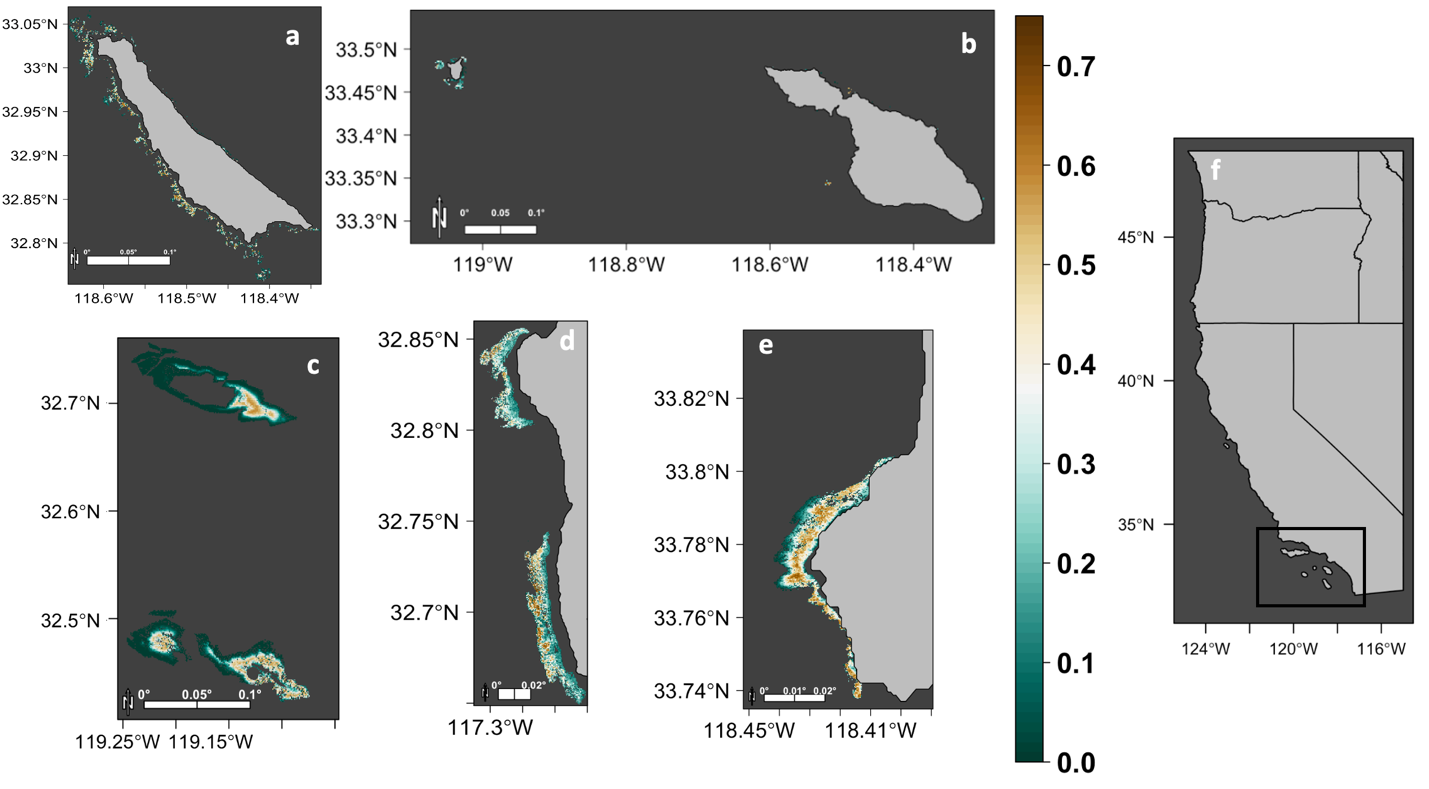


Figure 1. Predicted relative mean probability of white abalone presence in five areas located within the Southern California Bight (f), including San Clemente Island (a), Santa Catalina and Santa Barbara Islands (b), Tanner and Cortes Banks (c), San Diego (d), and Palos Verdes (e). Each panel represents the mean probability of abalone presence averaged over 100 model runs, ranging from unsuitable (0) to most suitable (0.7).


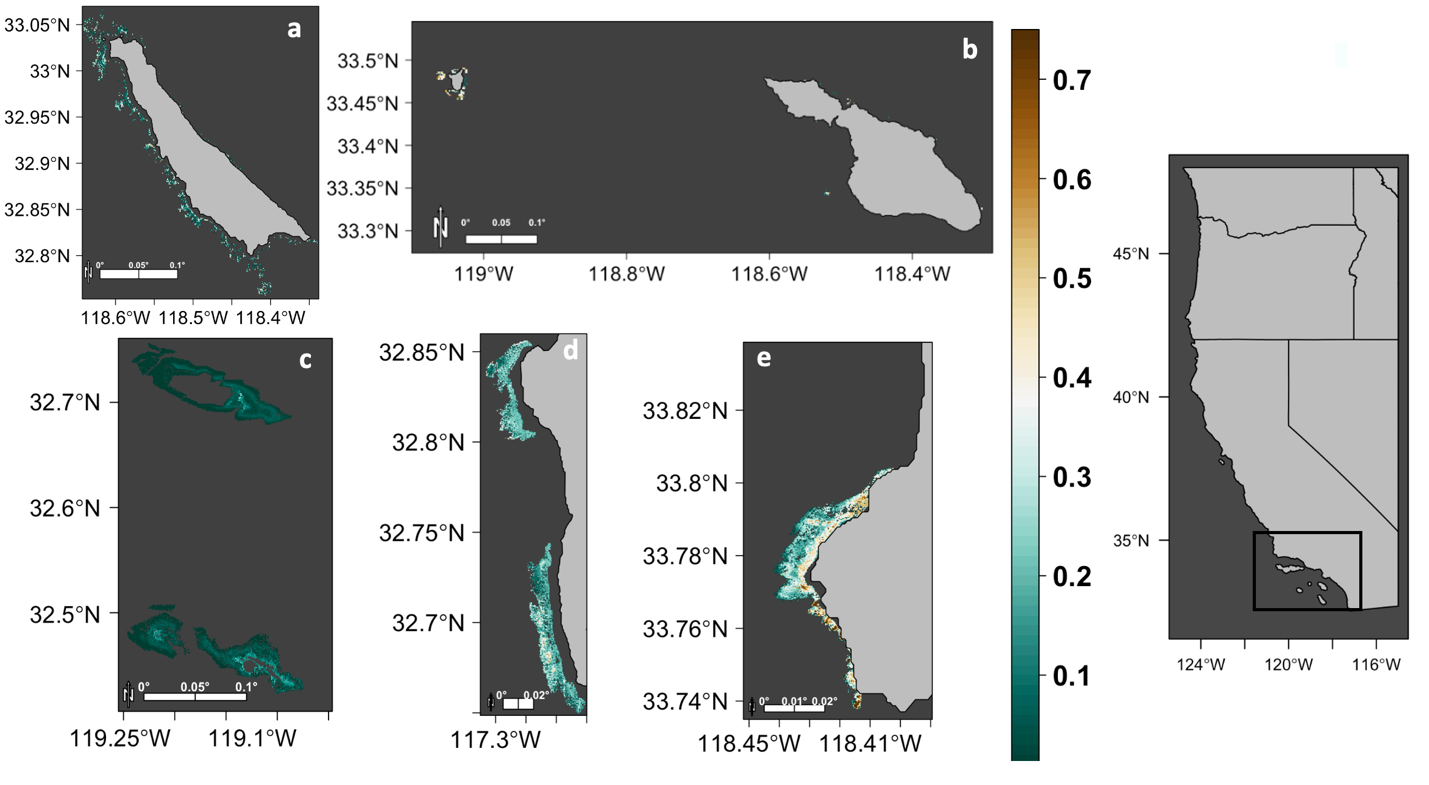


Figure 2. Error in model predictions of relative mean probability of white abalone presence in five areas located within the Southern California Bight (f), including San Clemente Island (a), Santa Catalina and Santa Barbara Islands (b), Tanner and Cortes Banks (c), San Diego (d), and Palos Verdes (e). Each panel represents the variability in predictions (95% quantile range), ranging from low variability (0) to high variability (1.0).


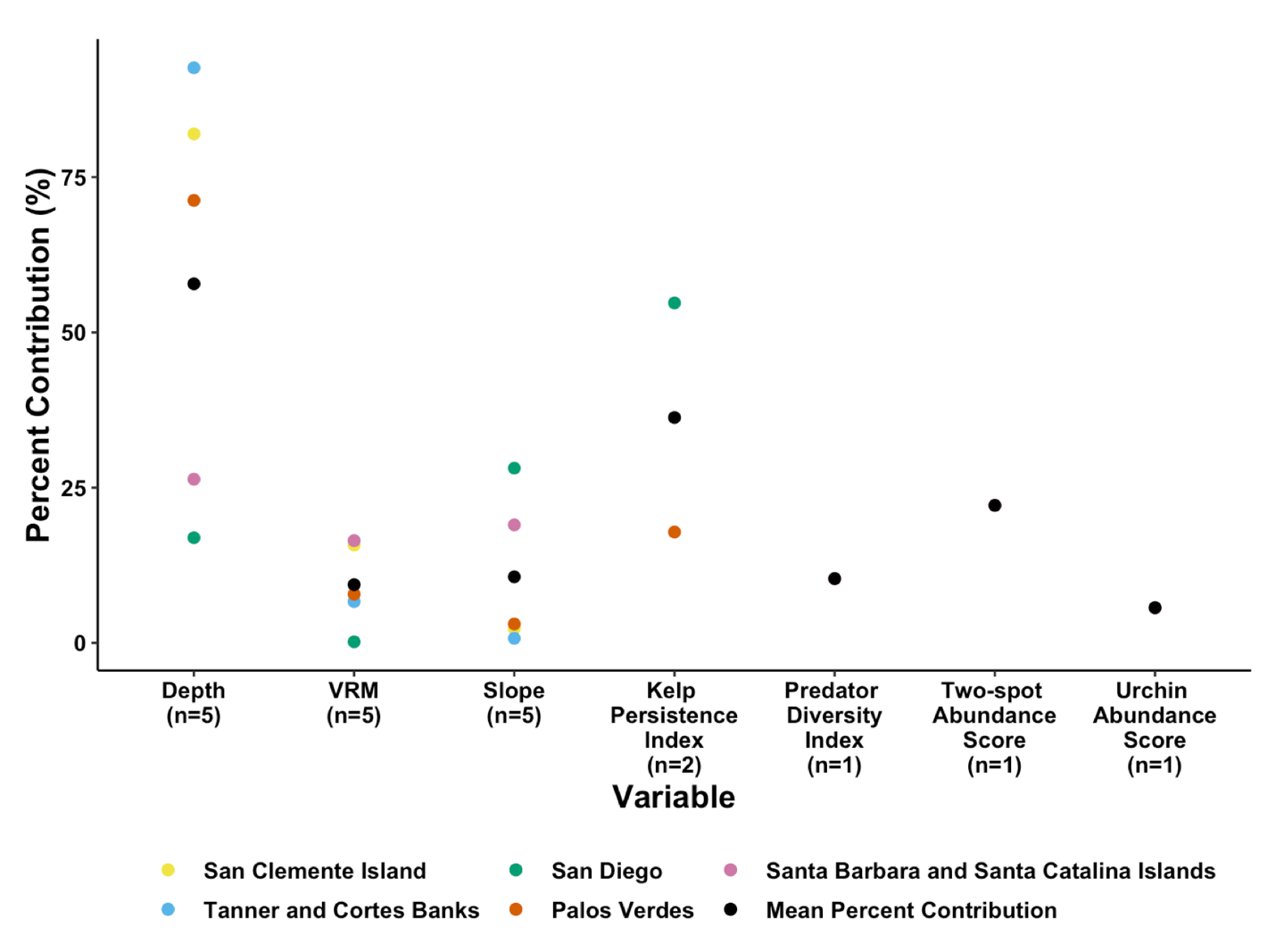


Figure 3. Percent Contribution (variable importance) by study area developed by MaxEnt using fine-scale, fishery-independent data. The black points represent mean variable importance averaged over study areas and n represents the number of study areas used to derive mean contribution for each variable.

Figure 4. Response curves for Depth (a), Slope (b), and VRM (c) developed by MaxEnt using fine-scale, fishery-independent data at San Clemente Island. The black line represents the mean response of the 100 model runs and the grey shaded region represents one SD of the mean.

Figure 5. Response curves for Depth(a), Slope (b), and VRM (c) developed by MaxEnt using fine-scale, fishery-independent data at Tanner and Cortes Banks. The black line represents the mean response of the 100 model runs and the grey shaded region represents one SD of the mean.

Figure 6. Response curves for Depth (a), Slope (b), VRM (c), Predator Diversity Index (d), Two-spot Abundance Score (e), and Urchin Abundance Score (f) developed by MaxEnt using fine-scale, fishery-independent data at Santa Barbara and Santa Catalina Islands. The black line represents the mean response of the 100 model runs and the grey shaded region represents one

SD of the mean.

Figure 7. Response curves for Depth (a), Slope (b), VRM (c), and Kelp Persistence (d) developed by MaxEnt using fine-scale, fishery-independent data at San Diego. The black line represents the mean response of the 100 model runs and the grey shaded region represents one SD of the mean.

Figure 8. Response curves for Depth (a), Slope (b), VRM (c), and Kelp Persistence (d) developed by MaxEnt using fine-scale, fishery-independent data at Palos Verdes. The black line represents the mean response of the 100 model runs and the grey shaded region represents one SD of the mean.
